# HIV-1 Latency and Viral Reservoirs: Existing Reversal Approaches and Potential Technologies, Targets, and Pathways Involved in HIV Latency Studies

**DOI:** 10.3390/cells10020475

**Published:** 2021-02-23

**Authors:** Sushant Khanal, Madison Schank, Mohamed El Gazzar, Jonathan P. Moorman, Zhi Q. Yao

**Affiliations:** 1 Center of Excellence in Inflammation, Infectious Disease and Immunity, James H. Quillen College of Medicine, East Tennessee State University, Johnson City, TN 37614, USA; khanals@etsu.edu (S.K.); niecem@etsu.edu (M.S.); ELGAZZAR@etsu.edu (M.E.G.); moorman@etsu.edu (J.P.M.); 2 Division of Infectious, Inflammatory and Immunologic Diseases, Department of Internal Medicine, Quillen College of Medicine, East Tennessee State University, Johnson City, TN 37614, USA; 3 Hepatitis (HCV/HBV/HIV) Program, James H. Quillen VA Medical Center, Department of Veterans Affairs, Johnson City, TN 37614, USA

**Keywords:** ART, HIV, latency, latency reversal, provirus, reservoir

## Abstract

Eradication of latent human immunodeficiency virus (HIV) infection is a global health challenge. Reactivation of HIV latency and killing of virus-infected cells, the so-called “kick and kill” or “shock and kill” approaches, are a popular strategy for HIV cure. While antiretroviral therapy (ART) halts HIV replication by targeting multiple steps in the HIV life cycle, including viral entry, integration, replication, and production, it cannot get rid of the occult provirus incorporated into the host-cell genome. These latent proviruses are replication-competent and can rebound in cases of ART interruption or cessation. In general, a very small population of cells harbor provirus, serve as reservoirs in ART-controlled HIV subjects, and are capable of expressing little to no HIV RNA or proteins. Beyond the canonical resting memory CD4^+^ T cells, HIV reservoirs also exist within tissue macrophages, myeloid cells, brain microglial cells, gut epithelial cells, and hematopoietic stem cells (HSCs). Despite a lack of active viral production, latently HIV-infected subjects continue to exhibit aberrant cellular signaling and metabolic dysfunction, leading to minor to major cellular and systemic complications or comorbidities. These include genomic DNA damage; telomere attrition; mitochondrial dysfunction; premature aging; and lymphocytic, cardiac, renal, hepatic, or pulmonary dysfunctions. Therefore, the arcane machineries involved in HIV latency and its reversal warrant further studies to identify the cryptic mechanisms of HIV reservoir formation and clearance. In this review, we discuss several molecules and signaling pathways, some of which have dual roles in maintaining or reversing HIV latency and reservoirs, and describe some evolving strategies and possible approaches to eliminate viral reservoirs and, ultimately, cure/eradicate HIV infection.

## 1. Introduction

### 1.1. HIV Reservoirs, Latency Maintenance, and Clinical Complications

HIV latency is a major problem that has been recognized since the introduction of combined antiretroviral therapy (ART) to inhibit viral replication and disease progression. Latently HIV-infected patients on ART may contain as low as one copy of provirus incorporated into the genome of host cells [1]. These proviruses remain silent in resting cells but are replication-competent, resulting in latent HIV reservoirs. To date, the mechanisms underlying HIV reservoir formation and the cell types involved in establishing HIV latency are not fully explicit. Recent studies suggest that these latent reservoirs are formed very early during the acute phase of HIV infection and accumulate over time [2]. Some studies also identify that latency establishes near the time of ART initiation, and some show a reduction of viral reservoir size in HIV-infected adults with early ART initiation [3,4,5,6,7,8]. Several types of cells, including memory CD4 T cells, dendritic cells, myeloid cells, epithelial cells, microglia, and even HSCs, have been identified as HIV reservoirs, harboring provirus [9,10]. The lymphoid tissues are the primary sites of viral replication, and active and prolonged clinical latency is initiated and maintained in these sites (lymph nodes, spleen, gut-associated lymphatic tissues), which are also the most active sites during viral infections given their role in immune-cell maturation/activation, and forming a major reservoir for HIV latency that has been characterized and discussed profoundly [11,12,13,14,15,16].

While several reviews have described strategies and methods to eliminate latent HIV reservoirs, compelling advances in this field are forthcoming. Of note, the classically described HIV receptor CD4, in conjunction with the coreceptors CCR5 and CXCR4, remain as canonical cellular receptors for HIV infection in all cell types in humans, with the exception of epithelial cells and brain astrocytes, which can be infected and harbor HIV provirus via syncytial fusion without any expression of the CD4 receptor on the cell surface [10,17,18,19]. Nevertheless, one major problem in this field is the unavailability of established cellular marker(s) to identify provirus-harboring reservoir cells. An extensive study on putative reservoir markers such as CD2, CD20, CD30, and CD32a has been published before [20]. A recent study identified CD4 T cells with a surface immunoglobulin marker—CD32a^+^, which contains a significant percentage (26.8 to 83.3%) of quiescent HIV proviral DNA [21]. However, another study reported that CD32a is more of an activation marker that is mainly presented in activated CD4 T cells showing a Th_2_ phenotype than a marker for resting CD4 T cells, and is coexpressed with HIV-RNA in activated cells in vitro and in vivo [22]. CD32a^+^ cells have distinctive phenotype from CD32a^-^ cells, but activation markers (HLA-DR^+^, CD69^+^, and CD25^+^) in blood and lymph nodes show no difference in both viremic and aviremic HIV^+^ subjects when compared to HIV^-^ subjects [22]. This is just one example demonstrating the complexity of identifying a resolute and orthodox latent HIV reservoir marker.

HIV latency in the era of ART is characterized by the existence of viral reservoirs that prevent HIV eradication and likely hinder complete immune reconstitution [23,24]. Latently HIV-infected cells generally do not actively produce viral particles under effective ART suppression; however, ART cessation predictably allows for rapid viral rebound [1,25,26,27]. Of note, active HIV production is not required to induce negative cellular effects, and the latent infection triggers numerous alterations in cellular signaling and metabolic dysfunctions, and thus leads to persistent inflammation and premature immune aging [28,29]. In particular, patients with latent HIV infection on ART exhibit both immunologic scarring and low-grade inflammation, inducing an inflammaging phenotype characterized by aberrant DNA damage and repair signaling, shortened telomeres, genomic instability, impaired mitochondrial functions, poor proliferative capacity, and blunted vaccine responses [28,29,30,31,32,33,34,35,36,37,38,39,40,41,42,43]. This inflammaging in the setting of ART-controlled, latent HIV infection exposes the immune system to unique challenges that lead to profound T cell exhaustion and senescence, a situation that drives increased infections, cancers, cardiovascular diseases, and neurodegeneration, similar to what is observed in the elderly [44,45]. Indeed, people living with HIV while on ART are prone to chronic renal disease, lung disease, cardiovascular complications, and mental disorders such as schizophrenia, depression, and anxiety [46,47,48,49,50]. This population also exhibits an increase in abnormalities associated with metabolic disorders, such as increased rates of obesity, high body mass index (BMI), and waist–hip ratios (WHR) [48,51]. In young children with HIV on ART, a fragile mitochondrial DNA (mtDNA) haplogroup H can lead to decreased mtDNA content, impaired mitochondrial markers, reduced complex IV activities, and overall growth retardation [52,53]. Mitochondrial dysfunction can also induce alterations in mitochondrial membrane potential, reactive oxygen species (ROS) accumulation, oxidative DNA damage, and aggregation of acetyl CoA, resulting in altered insulin resistance, lipid profiling, and hepatic steatosis [28,53,54,55,56]. Therefore, it is fundamentally important and clinically significant to identify, reverse, and eradicate HIV reservoirs in individuals with ART-controlled, latent HIV infection.

Eliminating HIV reservoirs and achieving HIV cure is very challenging. First, the host immune system cannot recognize and clear latently HIV-infected cells in the absence of detectable viral RNA or proteins. Second, there are no specific or widely accepted cellular latency markers (excluding the putative CD2, CD20, CD30, CD32a, and some immune checkpoint molecules) to target and clear the reservoirs in ART-controlled HIV subjects [20]. Third, while latency-reversal agents (LRAs) have been used to reactivate latently infected cells, clearance of these reactivated HIV-harboring cells is also very difficult to assess because of limited information on the markers of latency reversal; the regions/sites of provirus incorporation/integration within the cellular genome; and the role of a broad range of cells, tissues, or organs harboring these HIV reservoirs, which are usually immune-privileged and subject to immune evasion. The information available on integration sites varies highly between cell types, the techniques used to examine these data, and other experimental parameters. We currently do not have concrete information regarding the appropriate or canonical number(s) of integration sites. For instance, a total of 40,569 integration sites were revealed in Jurkat cells using a pyrosequencing technique [57]. Another study identified 6,719 integration sites in CD4 T cells in a study that included 13 individuals [58]. Genomic DNA sequencing identified the numbers of integration sites to be approximately 667–754, 589–649, and 577 in U1, ACH-2, and J1.1 cell lines, respectively [59]. Other studies identified integration sites in up to 2,661 locations in an in vitro primary CD4 T cell infection model, where integration favored active host transcription units, but with different integration preferences in activated and resting CD4 T cell types [60]. Integration sites also vary depending on treatments and stimulation conditions. For instance, using an inverse PCR and cloning technique, a CCL19-treated latency model in CD4 T cells revealed 247 integration sites, hosted by 85 genes; whereas PHA/IL-2 activated CD4 T cells had 432 integration sites hosted by 152 genes; and unactivated CD4 T cells had 133 integration sites hosted by 62 genes [61]. Another study carried out in CD4 T cells derived from 3 HIV patients identified 100 integration sites, of which 84 harbored a defective proviral sequence [62]. Given the arduous methodology and information about identifying the appropriate integration sites, eliminating HIV-1 latency based on integration sites appears to be very challenging.

Strategies to reverse HIV latency and potential techniques to “kick and kill” the reactivated, latently HIV-infected cells via inducing cellular apoptotic components have been reviewed previously [63]. To understand the broader aspect of latency eradication for an HIV cure, an extensive investigation of latency reversal approaches, as well as cell survival and apoptotic pathways under both latent and reactivated conditions, is an unmet medical need. Therefore, in this review we focus on discussing the strategies and approaches needed to identify poorly understood latency-reversal molecules/pathways and the mechanisms directly or indirectly involved in latency reversal and latent cell survival and/or longevity. The cellular components/mechanisms discussed here have shown involvement in either latency perseverance and/or reversal that could be the focus of future studies to unveil the complex mysteries of HIV latency.

### 1.2. HIV Latency and Reversal Approaches

In CD4 T cells, HIV reservoirs may be established due to either direct infection of activated memory T cells or progression of infected naïve or effector CD4^+^ T cells to a memory/resting T cell phenotype [1,64]. In several in vitro models of HIV latency, investigators have employed conditional culture media containing antibodies and cytokines (anti-IL-4 and anti-IL-12 antibodies, TGFβ1) and T cell receptor (TCR) stimuli (anti-CD3 and anti-CD28) to enhance naïve or effector T cell progression to memory T cells [30,65,66]. In these model systems, memory T cells are infected by HIV (such as the HIV-1_NL4-3_ strain) and then treated with or without ART and LRAs to study latency reversal and associated G-quadruplex-controlled gene expression or DNA damage response [30,65,66]. In this latent primary memory T cell model, researchers reported a higher degree of viral reactivation by agents such as bryostatin (62%) and ingenol 3, 20-dibenzoate (127%) relative to anti-CD3 and anti-CD28 stimulation, which were reduced to 15% and 22% when the cells were treated by SAHA and PAM3CSK4, respectively [65]. Meanwhile, treatment with PMA or prostratin, which directly activates protein kinase C, TNFα activating NF-κB, and the use of valproic acid to inhibit histone deacetylases (HDACs) failed to induce latency reactivation or viral gene expression [66]. Stimulation of calcium influx only using ionomycine had no effects on viral reactivation, but when ionomycine and PMA were used together, a minor reactivation (4%) was observed [66]. Some studies have shown that different cell types and LRA combination play a big role in triggering an effective viral reactivation [66,67]. The alcohol antagonist drug disulfiram can reactivate latency only in cell lines of myeloid origin (U1, THP89, and CHME5) but not in T-lymphoid cell lines (J-Lat 9.2, J-Lat A1 and A2); and a combination of romidepsin and disulfiram showed only minimal effects in reversing latency, both ex vivo and in vivo [67]. Not only cell types but also different mechanisms come to play in affecting the degree of latency reversion. In both Jurkat cell line (2D10) and in the primary CD4 T cellular model of HIV latency, a triple combination of three agents, procyanidin trimer C1 (a MAPK agonist), kansui (a PKC agonist), and JQ1 (a BET bromodomain inhibitor and pTEFb activator) successfully reactivated latency, which failed when they were used in similar concentrations individually [68]. Notably, procyanidin trimer C1 and kansui are plant derivatives [68]. Other approaches to reverse HIV latency include using CpG oligodeoxynucleotides, which activate toll-like receptor 9 (TLR-9), or long noncoding RNA (lncRNA) uc002yug.2, which affects Runt-related transcription factors RUNX1b/c and viral Tat gene expressions [69,70]. In HIV and simian immunodeficiency virus (SIV) latency-reversal studies in animal models, an IL-15 agonist (known as N-803) can robustly reactivate the virus (SIV/HIV) in ART-treated macaques and BLT (bone marrow–liver–thymus) humanized mice only under a CD8 T cell-depleted condition [71]. However, co-culture of human CD8^+^ T cells and latently infected primary CD4^+^ T cells under ART treatment eventually blocked the in vitro latency-reversal effects of an IL-15 agonist and potent latency-reversal agent N-803 [71]. In addition, activation of the noncanonical NF-κB signaling pathway using the apoptosis inducer AZD5582 induced HIV and SIV RNA expression in ART-suppressed BLT mice and macaques, respectively, affecting a wide range of tissues and cells (lymph nodes, thymus, bone marrow, liver, and lungs) [72].

It is becoming clear that HIV elimination by targeting the latent proviral reservoirs is an attractive but very challenging task to achieve because of the poor understanding of the mechanisms underlying HIV reservoir/latency establishment, maintenance, and reversal. For example, follicular helper T cells (T_FH_) expressing high levels of programmed cell death-1 (PD-1) are involved in HIV production during the acute phase of HIV infection, but the same population can also evolve into latent HIV reservoirs during the chronic phase of HIV infection following ART treatment [73,74,75]. PD-1 blockade using a monoclonal antibody (pembrolizumab) followed by the treatment with the LRA bryostatin induces the production of HIV-1 [76]. Surprisingly, conventional methods of T cell activation using TCR (anti-CD3, CD28), cytokine (IL-2, IL-1R, IL-15R, IL-7R, TNF-α), and mitogen (PMA, prostratin) stimulations are not required in the setting of PD-1 blockade in conjunction with the prostratin treatment alone [66,71,76].

Monocyte-derived dendritic cells (MDC_s_) present antigens—either HIV-1 antigens or cytomegalovirus (CMV) antigens—to autologous latent CD4 T cells containing a replication-competent provirus to facilitate HIV-1 latency reversal [77]. These MDC_s_ also induce cytotoxic T lymphocyte functions to kill the exposed targets/reversing cells [77]. The blockade of the CD40L/CD40 signaling pathway significantly reduces the phenomenon of latency reversal [77]. Cell–cell interactions between monocytes/dendritic cells and latently HIV-infected T cells are crucial in reversing latency. In contrast, a post-activation T cell latency model in contact with monocyte and anti-CD3 stimulation showed a reduction in virus expression [78]. Importantly, it has been reported that a preactivation latency model with pretreatment by chemokine ligand CCL19 in the presence of monocytes and anti-CD3 stimulation showed an increased viral activation levels, and it subsequently correlated with a reduction in frequencies of HIV DNA in the preactivation model, which was opposite in case of the postactivation latency model [78,79]. A previous study indicated that CCL19-treated resting CD4 T cells show an increased stability of HIV integrase, which allows subsequent integration and latency establishment, and this process depends upon functions of NFκB binding sites, Pin1, and HIV-LTR region [61]. In addition, deletion of NFκB binding sites and the HIV-LTR region showed no integration of HIV genome in CCL19-treated CD4 T cells, and inhibitors of PI3K and Ras/Raf/Mitogen-activated protein kinase/ERK kinase (MEK)/ERK signaling pathways restricted the HIV integration [61]. It should be noted that HIV-infected and proliferating T cells are killed at a significantly high rate during this activation process, further supporting the importance of investigating cell death and survival pathways, including some crucial biochemical ligands, in these models [78].

The sensing of the viral ssRNA genome by endosomal Toll-like receptors (TLRs), specifically TLR8, but not TLR7 or TLR9, promoted T cell differentiation to Th7 and Th17, increased cytokine production, enhanced CD4 T cell activation, and reversed latency in patient-derived T cells under ART treatment [80]. Thus, a system with co-culture of latent HIV reservoir cells with varieties of purified cell surface proteins, cytokine receptors, and GPCRs may be useful in understanding latency reversal. This co-culture approach should explore a wide range of cells and tissue types. This may provide important insights into the mystery of HIV latency, its maintenance, and reversal.

**Figure 1:**
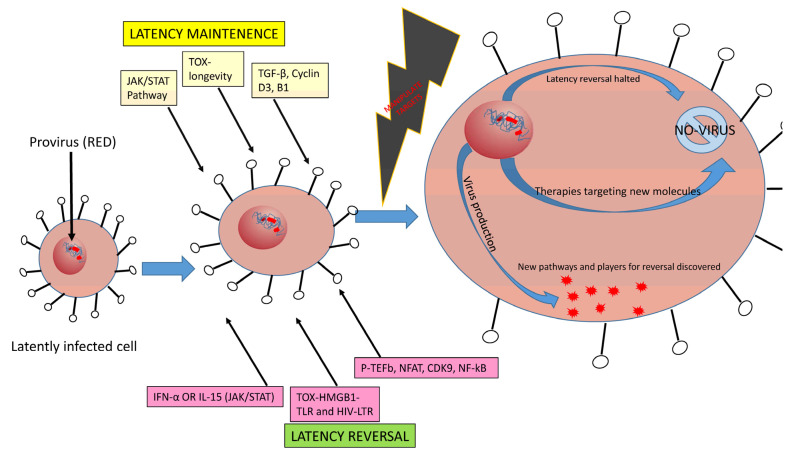
A model depicting the dual/mixed roles of signaling molecules/pathways involved in the maintenance/upkeep and/or reversal of HIV latency. Some of these molecules/pathways show effects in both reversion and/or upkeep, depending on the upstream/downstream molecules involved and compounds, agents, and molecules used during cell culture/stimulation conditions. Targeting these regulatory molecules/pathways with various agents, drugs, vaccines, or antibodies may lead to new therapeutics for viral reactivation and latency reversal, or perhaps even upkeep/maintenance. In addition, manipulating these individual effectors at the genetic or epigenetic level may also affect either latency reversal or maintenance. Components associated with effector protein family or signaling cascades such as JAK/STAT and TOX can play dual roles. Understanding the roles of these effectors, as well as their working conditions, may facilitate targeting those molecules/pathways to cure HIV.

Notably, the data on latency reversal using LRAs are somewhat controversial and not always consistent, especially for different types of reservoir cell populations [81]. In CD4 T cells, latency reversal varies in each subset, such as naive cells (T_NA_), stem cell memory (T_SCM_), central memory (T_CM_), transitional memory (T_TM_), effector memory (T_EM_), and terminally differentiated cells (T_TD_), likely due to the intrinsic differences between these cell populations. For example, the use of romidepsin and ingenol appears to be one of the more robust LRA combinations to increase latency reversal and produce HIV particles, but this combination is not uniformly effective for all T cell subtypes [81]. T_SCM_ cells are highly resistant to latency reversal by LRAs, except for treatment by a combination of panobinostat and Bryostatin-1 [81]. A canonical B lymphocyte antigen, CD20, is dimly expressed in CD4 T cells from HIV patients, which exhibit high activation and a memory phenotype. The expression of HIV-RNA is significantly higher in CD20^+^ CD4 T cells in both ART- suppressed and viremic patients [82]. In patients on ART, the use of the monoclonal antibody (mAb) rituximab against CD20 induces latent reservoir killing in combination with LRA [82]. Therefore, further studies of the distinct roles of these agents (LRAs, mAbs) that can focus on cell-surface markers, receptors, and proteins involved in cell-cycle regulation, latency maintenance, and reactivation could result in a breakthrough in HIV latency reversal. Figure 1 illustrates potential pathways and mediators involved in regulating HIV reservoir and latency maintenance, reactivation, and reversal.

## 2. HIV Latency and Potential Agents for Reversal

### 2.1. TOX in HIV Latency

Thymocyte selection-associated high-mobility group box (TOX) is an important player in T cell differentiation due to the presence of an adjacent lysine-rich region that may serve as a nuclear localization signal (NLS), facilitating TOX–DNA interactions and protein–protein interactions. The TOX gene family comprises four major isoforms (TOX1, TOX2, TOX3, and TOX4) in different chromosomal loci. TOX1 regulates immune differentiation, TOX2 regulates natural killer (NK) cells and is a DNA-binding transcription factor that functions in proximity to RNA polymerase II, TOX3 prevents neuronal death, and TOX4 is involved in the process of DNA damage repair and cell-cycle progression from mitosis to interphase [83,84,85,86,87,88,89,90]. Extremely high expression of TOX1 has been observed in microarray analysis of thymic transcripts in CD4^+^ CD8^+^ double-positive (DP) thymocytes [83]. This finding indicates the importance of TOX in immune cell differentiation. Furthermore, CD4 T cells, NK cells, and lymph nodes express high levels of TOX1. Failure of T cell and NK cell development and dysfunction of the transcription factor FOXP3 regulated gene expressions are observed in the absence of TOX1, further demonstrating its role in lymphocyte differentiation and functions [85]. Additionally, TOX is also significantly upregulated during tumor generation and progression [86]. A direct interaction between platinated DNA and TOX4 is required for DNA repair in cancer when platinating anticancer drugs, such as cisplatin, trigger DDR [88]. Surprisingly, on the other hand, some reports also suggest that TOX actually inhibits DNA repair by directly binding to KU70/80 and suppressing nonhomologous end joining (NHEJ) repair in T cell acute lymphoblastic leukemia (T-ALL), and when TOX was stably knocked down, it elevated NHEJ repair during DNA double-stranded breaks (DSB). TOX mutants lacking the NLS and high-mobility group (HMG) domain did not alter the NHEJ repair because NLS and HMG directly bind to KU70/80 via HMG domain [91]. These findings suggest the complexity and importance of TOX overall.

**Figure 2:**
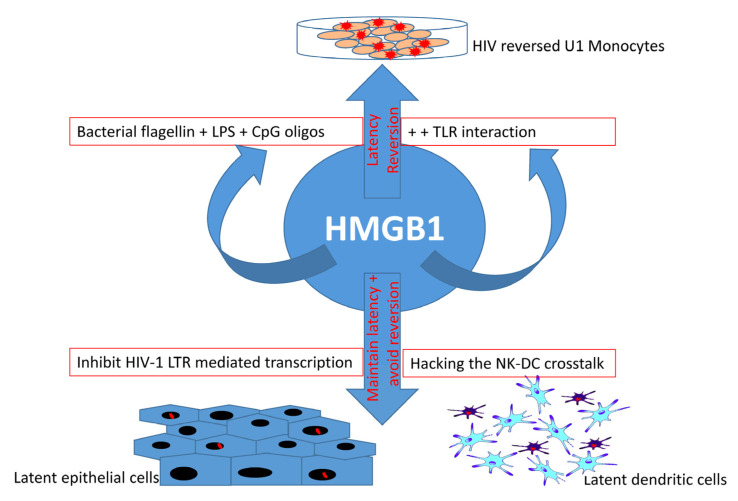
Dual role of HMGB1 in HIV latency. HMGB1 protein not only helps maintain viral latency in epithelial cells and dendritic cells, but also helps reverse HIV latency in U-1 monocytes. This reversal is associated with TLR interaction with bacteria-derived molecules (flagellin, LPS, and CpG oligos) that play a major role in HMGB1-associated reversal, suggesting the importance of foreign (bacterial) components in HIV reversal. However, as an indication of a dual role of HMGB1 protein family, latency is maintained and its reversion is prevented through the inhibition of HIV-1 LTR-mediated transcription and hacking the natural killer (NK) cells and dendritic cell (DC) crosstalk in latent epithelial cells and dendritic cells, respectively.

A significant decrease in HIV infectivity has been observed during an interplay of TOX4 and its interaction with the PWWP (Pro-Trp-Trp-Pro motif) interacting region (PIR) of a RNA binding protein and a splicing cofactor NOVA1 [92]. The PIR region of NOVA1 and the PIR region of TOX4 in TOX4 HMG domain interact with the PWWP domain of Lens epithelium-derived growth factor (LEDGF/p75), and when all three proteins (TOX4, NOVA1, and LEDGF) are localized in the nucleus and attached to the chromatin, they ultimately and specifically decrease HIV infectivity, but not murine leukemia virus (MLV) infectivity [92]. TOX and its association with high-mobility group box protein 1 (HMGB1) has been shown to play a role in HIV latency maintenance in dendritic cells via crosstalk with NK cells [93]. In epithelial cells, HMGB1 represses HIV replication by inhibiting long terminal repeat (LTR)-mediated transcription of the virion [94]. In contrast, the interaction between HMGB1 and TLR ligands is known to reverse HIV latency in chronically infected U-1 cells in the presence of bacterial components, such as flagellin, bacterial lipopolysaccharides (LPS), and CpG DNA oligos [95]. Importantly, TOX can bind to DNA in a sequence-independent manner to control transcription of a set of genes, such as CCR7, SELL, IL7R, NFAT5, LY6C1, CD38, CTLA4, LAG3, PDCD1, and many other genes associated with T cell longevity and maturation [96,97]. Thus, the dual role of the HMGB1 protein family and the widespread influence of TOX warrants a thorough investigation in order to elucidate their role in HIV latency maintenance and reversal (Figure 2). In a broader sense, TOX is transcriptionally, epigenetically, metabolically, and biochemically involved in T cell longevity, maturity, exhaustion, and survival. So far, there are no reports on specific compounds that can target TOX protein for HIV latency reversal. In silico analysis and molecular docking techniques identified a total of 140 compounds that bind the HMG box domain of the TOX protein, by virtual screening of 7.6 million agents from the ZINC15 database (118 identified) and screening of 200,000 other small molecules (22 identified) [98]. These 140 compounds were tested in vitro in a TOX-dependent Hut7b cell line model of cutaneous T cell lymphoma (CTCL), and 18 molecules were shown to inhibit TOX in both a TOX-high model (Hut78, SZ4, Jurkat cell lines) and a TOX-low model (K562, U937, and Mac2A) [98]. Antibodies and compounds that target HMG box have been reviewed in detail elsewhere, such as Anti-HMGB1 m2G7, acetylcholine, P5779, and resveratrol, which inhibit the signaling pathways associated with HMGB1 and TLR4 signaling [99,100,101]. Moreover, other HMG box protein-binding molecules, such as the receptor for advanced glycation products (RAGE), revealed a link between HIV infection and TLR4 signaling, and the use of RAGE/HMGB1 inhibitors such as FPS-ZM1 in latency study has already been evaluated [102,103,104,105]. Some of these molecules are potential candidates for future studies on HMG box protein and TOX-mediated HIV latency reversion or upkeep. Thus, mechanistic studies of TOX family proteins and their interacting components (ranging from chemicals compounds, antibodies, and RNA–DNA to protein–protein interactions) will help to understand the processes of immune-cell differentiation in long-lived, latently HIV-infected populations.

### 2.2. Protein Kinases in HIV Latency

Protein kinases and their roles in HIV latency/reservoir maintenance/reversal have been described previously [10,106]. A recent study using 418 structurally diverse, cell-permeable, and medicinally active kinase inhibitors in a latent cell line model showed that control of kinase activity can affect a wide range of cellular pathways or signaling cascades and block HIV-1 latency reversal [107]. One such multikinase inhibitor, midostaurin, plays a dual role by activating latency reversal and blocking viral replication or reversal in the presence or absence of SAM domain and HD domain-containing protein 1 (SAMDH1), respectively [108,109]. Kinome profiling recognizes the contribution of PIM-1 kinase in latent HIV infection and reactivation in T cell lines, as well as in primary CD4 T cells [110]. In both models, HIV reactivation is largely affected following inhibition or knockdown of PIM-1 by the PIM-1 inhibitor IV (PIMi IV) or PIM-1 specific shRNAs, respectively [110]. Derivatives of the compound benzolactam promote apoptosis in ACH-2 and J-lat cells via a protein kinase C (PKC)-induced latency-reversal pathway, and one such derivative (BL-V8-310) was found to have high LRA activity that also reduced cytotoxic cytokine secretion [111]. These studies reveal the prevailing importance of protein kinases in HIV-1 reservoir/latency maintenance and/or reversal. The individual kinase signaling pathways that are affected during HIV latency reversal have been described previously [107].

**Figure 3:**
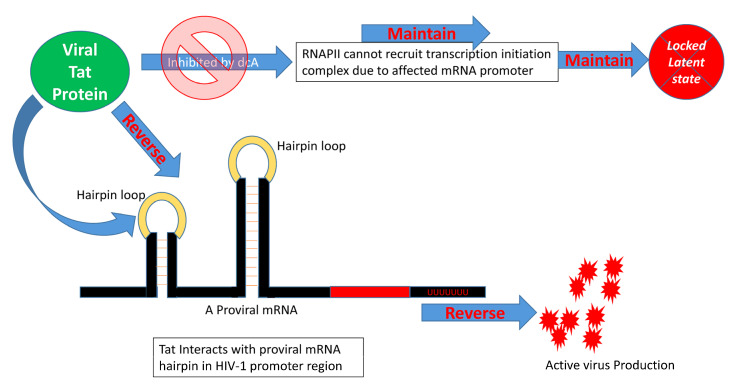
HIV Tat protein and latency reversal. Tat protein can reverse HIV latency by interacting with the promoter region of the HIV genome through recruiting RNAPII and associated transcription initiation complex. This process is affected by didehydro-cortistatin A (dCA). When RNAPII becomes unable to interact with the HIV mRNA hairpin loop, the latency reversal is halted. A thorough investigation of the roles of individual viral proteins in HIV latency reversal is thus very important for understanding their critical role in HIV cure.

The mammalian target of rapamycin (mTOR) protein is a serine/threonine protein kinase that belongs to the PI3K-related kinase family and is associated with a wide range of cellular processes such as cell proliferation, motility, growth, and survival, as well as protein synthesis and gene transcription. The interactions between dendritic cells and T cells through cell surface receptors/ligands, which activate PI3K-Akt-mTOR signaling by triggering dephosphorylation of proteins downstream of the Akt signaling pathway [112], play an important role in HIV latency reversal. However, such activation does not increase the expression/activity of nuclear transcription factors, such as NF-κB. Even blockade of c-Fos and c-Jun transcription factors, which directly bind to HIV-1 LTR promoter regions, does not affect dendritic cell contact-mediated latency reversal [112]. Also, HIV Tat drives latent provirus to an active virus-producing state by recruiting pTEFb complex and interacting with the viral mRNA hairpin in the HIV promoter region [113]. Cyclin T1 and CDK9 form the pTEFb protein complex to act with Tat as a cofactor for Tat-mediated transcription [114]. The CDK9 partner, cyclin T1, specifically enhances binding of Tat protein in the *trans*-activation response element (TAR) RNA stem loop structure [115,116,117]. The 42 kDa kinase CDK9, when mutated/inhibited, blocks Tat transactivation without exhibiting any effects in the overall T cell activation, which proves that CDK9 kinase activity is essential for Tat activation, which subsequently affects pTEFb recruitment and TAR RNA association [117,118,119,120,121,122]. Moreover, the amount of CDK9 phosphorylation and kinase activity can have direct effects on Tat association, and the degree of latency establishment; also, site-directed mutations within different locations within Tat protein residues revealed the importance of certain sites in the CDK9-Tat interplay in a crystallized model and in silico analysis of Tat-CDK9-Cyclin T complex [123,124]. The Tat inhibitor didehydro-Cortistatin A (dCA) can prevent the HIV transcription by blocking the assembly of Tat in HIV promoter with specific transcription factors and RNA polymerase II (RNAPII)-associated proteins, preventing the assembly of the transcription initiation complex, which eventually drives the cell toward a latent state (Figure 3) [113]. However, suppressing the multifunctional mTOR protein activity using mTOR inhibitors minimizes latency reversal in both a Tat-dependent and a Tat-independent manner by blocking CDK9 phosphorylation [114], stressing the importance of mTOR in latency. Another transcriptional regulatory factor, KAP1, is critical for the reactivation of HIV latency, which recruits CDK9 and interacts with the proviral promoter region allowing viral transcription [125]. Surprisingly, HIV-host transcription mechanisms have developed a bypass machinery to avoid KAP1-mediated activation in order to reduce the magnitude of virus production and latency reactivation [125]. Investigating the interplay between CDK9, KAP1, mTOR, and Tat may enhance our understanding of the HIV latency.

### 2.3. JAK/STAT Pathway in HIV Latency

A widely studied kinase cascade—the Janus kinase (JAK) signal transducer and activator of transcription (STAT) pathway (Figure 1)—has an important role in immune-cell activation, polarization, cytokine signaling, innate and adaptive immune responses, and autoimmune disease development [126,127,128,129]. The function of JAK/STAT in maintaining CD4 T cell progression has been reviewed previously [126]. Since latent reservoirs largely comprise CD4^+^ helper T cells, which have dual roles in acute and chronic HIV infection, investigating the role of the JAK/STAT pathway in establishing and maintaining the HIV reservoir in helper T cells is essential for increasing the understanding of their reversal [73,74,75,126,127,128]. The activation of JAK/STAT and the central regulator mTOR pathways inhibit HIV reactivation and prevent the production of virus particles from a latent state. However, inhibition of the JAK/STAT pathway promotes HIV-1 reversion [130]. In contrast, when JAK is inhibited using JAK inhibitors, such as tofacitinib and ruxolitinib, inhibition of viral production is observed in HIV reservoirs via suppression of an IL-15-mediated viral reactivation pathway [131,132]. These studies highlight the dual and complex role of the JAK/STAT pathway in HIV latency and reversal (Figure 1), and present this molecular pathway as a mechanism that warrants further investigation. STAT plays an important role in HIV development independent of the infection status (acute or chronic), and a use of benzotriazoles reactivates HIV latency by preventing a negative feedback loop carried out by SUMO2/3 (affecting phosphorylated STAT5), which sustained the STAT5 phosphorylation and its active form [133,134,135]. In addition, HIV-1 expression was restricted in U-1 latent cell lines following a heterodimer complex formation between p50 (viral promoter binding protein) and naturally occurring C-terminally truncated STAT5 [136]. The availability of STAT in its active form is important for latency reversion. On the other hand, STAT3 inhibitor 5,15-DPP at 50 and 500 nM has been shown to promote HIV-1 transmission and reversion when compared to 5 nM and no treatment using an envelope defective mutant HIV strain [130,137]. However, the STAT1 inhibitor fludarabine was shown to block IL-6 and HIV-1 interplay, reducing the monocyte migration and damage in a recent study [138]. Phosphorylation of STAT 1, 3, and 5 by IFN-α, but not others (IFN-β, ω, ε, λ1, and λ3) was successful in reversing HIV latency using in vitro cell models, as well as in CD4 T cells derived from patients undergoing ART [139]. The study of specific viral components in relation to the JAK/STAT pathway is essential to identify new targets for HIV latency reversal. For example, the viral accessory protein Vif is directly involved in the degradation of the JAK/STAT pathway [140]. Vif interacts with STAT1 and STAT3, but not STAT2, and plays a critical role in the prevention of the antiviral effects of Type-1 IFN-α signaling [140]. Therefore, the roles and mechanistic effects of LRAs, protein interactions, IFN activities, and cytokine signaling on the JAK/STAT pathway and on viral proteins should be investigated in detail in order to understand their potentials as molecular targets in HIV latency maintenance and/or reversal.

### 2.4. Apoptotic Proteins in HIV Latency

Productive viral infection, latency establishment, and latency reversion affect cellular metabolism. Cellular metabolism is hijacked by HIV-1 and changes in antioxidation, iron metabolism, and iron import are observed during latency transitions, while oxidative stress is increased and antioxidant response is upregulated during latency reversion and also during productive in vitro and in vivo infections [141]. Drugs increasing oxidative stress or iron content and an increase in antioxidant gene expression resulted in reactivation of latency, causing degradation of promyelocytic leukemia protein nuclear bodies [141]. Another drug, auranofin, which is used to treat rheumatoid arthritis, had an impact on the latent viral reservoir by reducing the number of the integrated viral DNA in HIV patients under ART [142]. Partial inhibition of glycolysis using 2-deoxy glucose blocked HIV-1 infection, decreased cell viability of preinfected cells, and most importantly, avoided latency reversion in CD4 T cells obtained from HIV patients under ART [143]. Uninfected and HIV-infected macrophages use fatty acid and glucose as primary sources of energy, however, latent macrophages used glutamine/glutamate as a major source of energy, and blocking the glutamine, glutamate, and alpha-ketoglutarate pathways killed latent HIV-infected macrophages [144]. Viral infections alter, hack, and reprogram host metabolism regularly, as discussed before [145,146]. Thus, there is a strong connection between latency upkeep/reversion and metabolic pathways, agents, drugs, etc.

**Figure 4:**
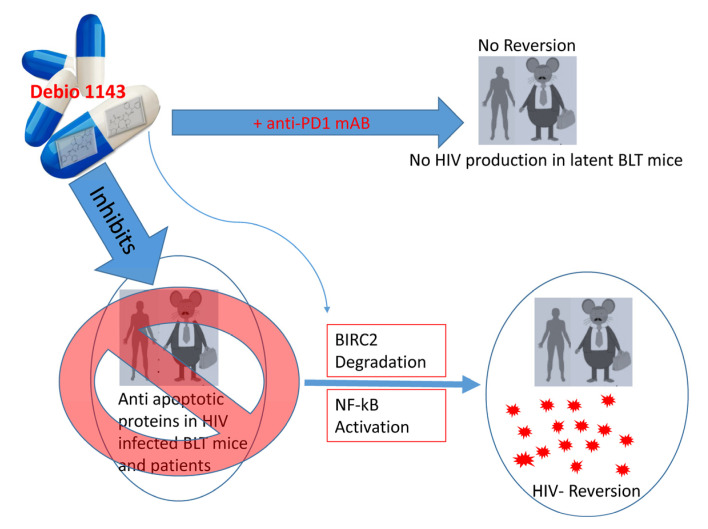
Inhibition of anti-apoptotic proteins reverses HIV latency. Debio 1143 (an IAP inhibitor) inhibits anti-apoptotic proteins such as BIRC2, which activates NF-κB-mediated HIV reversal. HIV latency can be markedly reversed in ART-treated BLT mice and human subjects when treated with Debio 1143. On the other hand, Debio 1143, when combined with anti-PD1 monoclonal antibody (mAb), can reduce HIV production and latency reversion in BLT humanized mice in most tissues (spleen, lymph nodes, liver, lungs, and thymic organoids). The dual role of chemical agents in HIV reversal/maintenance is thus intriguing and warrants further investigation.

Viral proteins such as Nef, Tat, Vpu, GP120, and Vpr have been shown to promote or inhibit cell apoptosis [147]. Many of the differential regulatory effects of these proteins are dependent upon the phase of HIV in infected cells. For instance, many of these proteins are anti-apoptotic during acute infection to enable persistent infection, but may transition to pro-apoptotic in the process of establishing chronic infection by inducing bystander apoptosis. The role of the envelope and protease proteins in activating apoptosis has been reviewed elsewhere [63,147]. Intriguingly, some viral proteins can have dual roles, with both pro- and anti-apoptotic effects [63,147]. Downregulation of pro-apoptotic proteins and/or upregulation of anti-apoptotic proteins are key to escaping apoptosis, and thus favor survival of latent cells. For example, Debio 1143 is an inhibitor of anti-apoptotic proteins that activates HIV transcription via NF-κB signaling by degrading BIRC2 protein that mediates anti-apoptotic effects on latently HIV-infected cells [148]. Figure 4 illustrates how Debio 1143 can reverse HIV-1 latency in resting CD4 T cells derived from peripheral blood mononuclear cells (PBMCs) of HIV patients and humanized BLT mice on ART treatment [148]. On the other hand, Debio 1143, in combination with an anti-PD1 mAb, exhibits a significant PD-1 blockade-mediated HIV reduction in all tissues (spleen, lymph nodes, liver, lungs, and thymic organoids) in BLT mice [149]. Enhanced expression of the anti-apoptotic protein BIRC5 and its upstream regulator OX40 in productively and latently-infected CD4 T cells promotes HIV-infected cell survival [150], whereas inhibition of these molecules enhances HIV-infected cell death [150].

The tumor suppressor protein p53 can eliminate HIV-infected cells by enhancing the expression of PTEN—a negative regulator of protein kinase B/AKT [63,151,152]. AKT couples with PIP-3 of the PIK3 signaling pathway and activates itself, which inhibits pro-apoptotic proteins while also coactivating anti-apoptotic proteins, such as pBad, Bcl-2, and FOX01 transcription factor [63,153,154]. Triggering receptor expressed on myeloid cells 1 (TREM1) silencing in HIV-infected macrophages reduces anti-apoptotic protein expression, increases pro-apoptotic signaling, and affects the survival of HIV-infected cells, leading to disruption of the mitochondrial membrane potential, cytochrome-C release, and caspase-9 cleavage [155]. HIV promotes latent cell survival in a TREM1-dependent fashion and manipulates the anti-apoptotic Bcl-2 protein family [155]. Thus, these signaling components can promote latent cell survival and HIV reservoir establishment. Interactions between HIV-1 and the Bcl-2 protein family, as well as possible therapeutics, have been reviewed elsewhere [156]. A detailed investigation of these pro- and anti-apoptotic proteins in latency establishment and maintenance is essential to develop novel reversal approaches [63]. New studies on the association between the apoptosis-related proteins and HIV latency may lead to better approaches to controlling their expression levels or developing new drugs to regulate their expression, either individually or in combination (e.g., Debio 1143 in combination with anti-PD1 mAb) (Figure 4) [149]. Additionally, proteins associated with cell-death mechanisms other than apoptosis during viral infection, such as autophagy (LC3B, SQSTM1/p62), pyroptosis (Caspase1, 3), ferroptosis (GPX4), and necroptosis (RIP1, 3), should be investigated in order to understand their roles in HIV latency maintenance and/or reversal [157,158,159,160,161,162,163,164,165].

### 2.5. Transcriptional and Genetic Factors in HIV Latency

In the CCL19 and IL-7 treated resting CD4 T latency model described above, an increase in the levels of three microRNAs (miRs) such as miR98, miR4516, and miR7974, was shown by next-generation sequencing, however inhibiting these miRNAs did not reverse latency [79]. It is clear that inhibiting these miRNAs might not only reverse latency, and more mechanisms/aspects are involved in it. However, the fact that these miRNAs were upregulated in these latent HIV models should not be ignored, and thus further studies of these noncoding genetic elements and other potential regulators seem to be essential for understanding the mechanisms of HIV latency. Another miRNA known as TAR was abundant in exosomal vesicles in supernatants collected from an in vitro infection model, as well as serum, cerebrospinal fluid, blood plasma and even in saliva from patients under ART and in serum from HIV-1 infected humanized mice [166,167,168,169]. During HIV latency, the incorporated proviral genome is extremely low (only 1-5 copies), but TAR RNA copy numbers range from 10^3^ to 10^5^ in patients undergoing ART suggesting that a true/complete transcriptional latency is not always the case [167,170]. As described previously, TAR RNA is essential for HIV Tat and protein kinase(s) RNA-activated (PKR) binding, however, the TAR miRNA binds to TLR7/TLR8, but not PKR [167]. Coculturing exosomal vesicles containing Tat, TAR RNA, TAR miRNA, and a newly found TAR-gag RNA induced IL-6, TNFβ, NF-κB pathways, cytokine production, and overall cellular activation of the recipient/neighbor cells, ultimately causing latency reversion [167,168,170,171]. These TAR RNA-containing exosomes also induced cancer-cell proliferation and progression by affecting expression of proto-oncogenes and TLR3 inducible genes, which reduced apoptosis in neighboring latency-reversing cells by lowering Bim and CDK9 protein levels [166,172]. These exosomal vesicles also contain phosphorylated c-Src, which causes PI3K-mTOR-AKT-mediated and P300/SRC-1, STAT3-activated latency reversion [171]. In addition, high levels of NF-κB and P300 were observed in the nuclei of latent cells cocultured with exosomal vesicles [171]. These vesicles can be targeted with antibodies, antibiotics, drugs, transcription inhibitors, and ARTs to alter the ratio/amount of their individual components and subsequently alter their roles in HIV latency upkeep/reversion [173,174]. For example, ARTs (Indinacir and Emitricitabine), antibiotics (oxytetracycline, tetracycline, methacycline, and demeclocycline), and even interferon treatments showed effects on the proteins involved in the endosomal sorting complex required for transport (ESCRT) pathway and caused changes in TAR RNA levels and other contents of these exosomal vesicles [173]. In addition, incorporation of a transcriptional inhibitor F07#13 in a mathematical model in various cell types displayed potential to induce changes in HIV latency and LTR dynamics and were different among the cell types used [174]. In a study that used a library of FDA-approved drugs, HIV-1 proviral transcription was activated with febuxostat, eltrombopag, and resveratrol, while mycophenolate inhibited HIV-1 proviral transcription, and these transcriptional modulators exhibited different effects in different cell types (lymphoid versus myeloid lineage) [175].

A newly identified QUECEL (quiescent effector cell latency) model used polarized T cell subsets (Th1, Th2, Th17, and T_REG_) to mimic HIV latency in vitro [176]. This model represents the escape of latently infected cells from cell-cycle checkpoints and showed a significant reduction in cell-cycle-dependent cyclins D3 and B1, and restricted expression of cell-activation markers such as CD69 and CD25, and the positive transcription elongation factor P-TEFb (Figure 1) [176]. RNA-Seq and follow-up studies in quiescent cells suggests that altered gene expression is associated with latent cell expansion, reinforcing the roles of cyclin-dependent kinase pTEFb induction and CDK9 phosphorylation in latency reactivation and the anti-inflammatory molecule TGF-β elevation in latency maintenance [176,177,178]. Notably, the QUECEL model revealed alterations in regulatory machineries, indicating that the c-Myc pathways are highly repressed and NF-κB is largely dispensable, whereas nuclear factor of activated T cells (NFAT) and NFAT-dependent latency reactivation are required for HIV latency [176]. NF-κB and NFAT binding at sites in the proviral enhancer are calcium-dependent and positively regulate viral transcription as well as T cell activation, and the NF-κB, NFAT, when bound can recruit a histone acetyltransferase, p65, in LTR regions to promote HIV transcription [179,180,181,182,183,184].

The HIV genome promoter regions U3 and R, which have a greater involvement of the 5′-long terminal repeat (LTR) section and a minimal participation of the 3′-LTR section, are responsible for controlling viral expression following different mechanisms of transcriptional interference in these regions of the viral genome, such as promoter occlusion and steric hindrance, because the viral DNA blocks the active and normal transcriptional process within the region of its incorporation in the host-cell chromosome [185,186,187,188,189]. The 5′-LTR region is very important in controlling transcription because the interaction between negative elongation factor (NELF) and RNAPII can lead to premature termination of transcription to limit the escape of the transcriptional complexes [185,186,187,188,189,190,191]. The mechanisms involved in controlling histone deacetylation and methylation, along with the use of long noncoding RNAs (lncRNAs) to repress the latency reversal, has been reviewed elsewhere [185]. Previous studies showed that a single nucleotide polymorphism (SNP) in a genomic region with close proximity to the CCR5 coding region could control HIV-1 associated coreceptor CCR5 mRNA expression via CCR5AS-lncRNA-mediated sequestering of Raly, a protein that binds and degrades CCR5 mRNA [192]. The reduction in CCR5-lncRNA failed to protect the CCR5-mRNA and resulted in a very low expression of CCR5 on the cell surface [192]. The ability to manipulate the expressions of these coreceptors during acute/chronic stages of viral infection could have an effect on HIV latency. Another SNP (rs2027820) with a virtually perfect linkage disequilibrium with the previous (rs1015164) SNP also controls the coreceptor CCR5 through differential binding of activating transcription factor (ATF1) [192]. These studies imply that future research should focus on such noncoding elements, polymorphic areas, SNPs, etc. The importance of the genomic noncoding regions in HIV pathogenesis could be a new area of investigation, which should focus on the role of these noncoding RNA sequences during latency initiation, maintenance/upkeep, and reversal [192].

Notably, the mechanisms associated with the host mRNA decay and the proteins involved in active HIV infection are quite different from those involved in latent HIV infection. The host mRNA decay proteins UPF1, UPF2, SMG6, and Staufen1 were significantly downregulated in monocyte-derived macrophages (MDMs) infected with HIV [193]. UPF2 and SMG6 downregulation via siRNA-mediated silencing enhances HIV gene expression; however, Staufen1 silencing impairs HIV gene expression [193]. Further investigation of the viral proteins involved in these cellular defense machineries is warranted and could uncover more molecular and genetic targets needed for HIV cure.

## 3. Conclusions and Perspectives

A thorough understanding of the mechanisms of HIV latency is critical for the development of clinical approaches and technologies for HIV latency reversal and cure. Identifying novel pathways or effector molecules involved in latency maintenance and reversal is appealing (Figure 1). New findings regarding pathways related to cell longevity and immune-cell differentiation or regulatory kinase pathways and their control over transcription factors and cytokine expression during HIV latency are crucial [78,79,112]. Therefore, the involvement of pro- and anti-apoptotic proteins and their interactions with viral proteins in immune cells during latent HIV infection warrants further investigation. In particular, new research should focus on the transcription initiation complex formation and the recruitment of various transcription factors and associated cofactors to the LTR promoter region of the proviral DNA, which may serve as molecular targets for a future HIV cure.

Because HIV reservoirs often hide in immune-privileged locations such as lymphatic, gut, or brain tissues, researchers have developed techniques using nanoparticle-packed ART drugs (nanomedicines), including the base-editing CRISPR-Cas9 system that can directly target the provirus and eliminate HIV reservoirs [25]. These gene-editing techniques in combination with latency reversal or reservoir reactivation effectors/molecules can be employed to target and “chop off” the proviruses, and may lead to a breakthrough toward HIV cure. Additionally, identification of novel latency-associated molecules may reveal potential targets for drug and vaccine developments. This form of treatment or prevention, if successful, will be easy to deliver and affordable in developing and developed countries. Hence, a more in-depth investigation with a focus on the latency reversal pathways in latently HIV-infected models is strongly encouraged.

Finally, the major obstacle in HIV-1 eradication is the multilayered mechanism of the establishment of HIV latency, as well how as the latent reservoir rebounds and produces infectious HIV virions in the setting of ART cessation. But even with successful control of viral replication, ART-controlled HIV subjects exhibit multiple signs of DNA damage, DNA repair inaccuracy, and mitochondrial dysfunction, leading to inflammaging, which is a major driver for a wide range of clinical complications. These individuals show minor to major complications involving various organ tissues such as lungs, liver, heart, kidneys, and brain, leading to premature aging and degeneration. Thus, there is an urgent need to develop a novel approach to eliminate HIV reservoirs even in the era of ART. To achieve this, a better understanding of how latency is established and the factors associated with HIV latency maintenance and reversal is essential. Additionally, a combination of latency-reversal methods based on understanding the signaling pathways/molecules and innovative gene-editing techniques targeting these components represent a potential leap in HIV gene therapy. This review supports mainstream efforts for identifying molecular pathway targets and therapeutic modalities related to latency upkeep and reversal to cure HIV.

## Abbreviations

ART	Anti-retroviral therapy
HIV	Human immunodeficiency virus
HSCs	Hematopoietic stem cells
LRA	Latency-reversing agents
HMG	High-mobility group
TCR	T cell receptor
LncRNA	Long noncoding RNA
BLT	Bone marrow, liver, and thymus
MDCs	Monocyte-derived dendritic cells
TLRs	Toll-like receptors
NLS	Nuclear localization signal
NHEJ	Nonhomologous end joining
PIR	Pro-Trp-Trp-Pro motif interacting region
TOX	Thymocyte selection-associated high-mobility group box
HMGB1	High-mobility group box 1
LPS	lipopolysaccharides
mTOR	Mammalian target of Rapamycin
JAK	Janus kinase
STAT	Signal transducer and activator of transcription
PD-1	Programmed cell death-1
QUECEL	Quiescent effector cell latency
LTR	Long terminal repeats
RNAPII	RNA polymerase II
SNP	Single nucleotide polymorphisms
TREM1	Triggering receptor expressed on myeloid cells 1
mAb	Monoclonal antibody
dCA	didehydro-Cortistatin A
NK	Natural killer

## References

[1] Siliciano R.F., Greene W.C. (2011). HIV Latency. Cold Spring Harb. Perspect. Med..

[2] Vanhamel J., Bruggemans A., Debyser Z. (2019). Establishment of latent HIV-1 reservoirs: What do we really know?. J. Virus Erad..

[3] Ananworanich J., Dubé K., Chomont N. (2015). How does the timing of antiretroviral therapy initiation in acute infection affect HIV reservoirs?. Curr. Opin. HIV AIDS.

[4] Leite T.F., Delatorre E., Côrtes F.H., Ferreira A.C.G., Cardoso S.W., Grinsztejn B., De Andrade M.M., Veloso V.G., Morgado M.G., Guimarães M.L. (2019). Reduction of HIV-1 Reservoir Size and Diversity After 1 Year of cART Among Brazilian Individuals Starting Treatment During Early Stages of Acute Infection. Front. Microbiol..

[5] Luo L., Wang N., Yue Y., Han Y., Lv W., Liu Z., Qiu Z., Lu H., Tang X., Zhang T. (2019). The effects of antiretroviral therapy initiation time on HIV reservoir size in Chinese chronically HIV infected patients: A prospective, multi-site cohort study. BMC Infect. Dis..

[6] Brodin J., Zanini F., Thebo L., Lanz C., Bratt G., Neher R.A., Albert J. (2016). Establishment and stability of the latent HIV-1 DNA reservoir. eLife.

[7] Pankau M.D., Reeves D.B., Harkins E., Ronen K., Jaoko W., Mandaliya K., Graham S.M., McClelland R.S., Iv F.A.M., Schiffer J.T. (2020). Dynamics of HIV DNA reservoir seeding in a cohort of superinfected Kenyan women. PLoS Pathog..

[8] Abrahams M.-R., Joseph S.B., Garrett N., Tyers L., Moeser M., Archin N., Council O.D., Matten D., Zhou S., Doolabh D. (2019). The replication-competent HIV-1 latent reservoir is primarily established near the time of therapy initiation. Sci. Transl. Med..

[9] Kuo H.-H., Lichterfeld M. (2018). Recent progress in understanding HIV reservoirs. Curr. Opin. HIV AIDS.

[10] Sadowski I., Hashemi F.B. (2019). Strategies to eradicate HIV from infected patients: Elimination of latent provirus reservoirs. Cell. Mol. Life Sci..

[11] Pantaleo G., Graziosi C., Demarest J.F., Butini L., Montroni M., Fox C.H., Orenstein J.M., Kotler D.P., Fauci A.S. (1993). HIV infection is active and progressive in lymphoid tissue during the clinically latent stage of disease. Nat. Cell Biol..

[12] Pantaleo G., Graziosi C., Butini L., Pizzo P.A., Schnittman S.M., Kotler D.P., Fauci A.S. (1991). Lymphoid organs function as major reservoirs for human immunodeficiency virus. Proc. Natl. Acad. Sci. USA.

[13] Pantaleo G., Graziosi C., Fauci A.S. (1993). The role of lymphoid organs in the pathogenesis of HIV infection. Semin. Immunol..

[14] Josefsson L., Palmer S., Faria N.R., Lemey P., Casazza J., Ambrozak D., Kearney M., Shao W., Kottilil S., Sneller M. (2013). Single Cell Analysis of Lymph Node Tissue from HIV-1 Infected Patients Reveals that the Majority of CD4+ T-cells Contain One HIV-1 DNA Molecule. PLoS Pathog..

[15] Pantaleo G., Graziosi C., Demarest J.F., Cohen O.J., Vaccarezza M., Gantt K., Muro-Cacho C., Fauci A.S. (1994). Role of Lymphoid Organs in the Pathogenesis of Human Immunodeficiency Virus (HIV) Infection. Immunol. Rev..

[16] Wong J.K., Yukl S.A. (2016). Tissue reservoirs of HIV. Curr. Opin. HIV AIDS.

[17] Chinnapaiyan S., Parira T., Dutta R., Agudelo M., Morris A., Nair M., Unwalla H.J. (2017). HIV Infects Bronchial Epithelium and Suppresses Components of the Mucociliary Clearance Apparatus. PLoS ONE.

[18] Winston J.A., Bruggeman L.A., Ross M.D., Jacobson J., Ross L., D’Agati V.D., Klotman P.E., Klotman M.E. (2001). Nephropathy and Establishment of a Renal Reservoir of HIV Type 1 during Primary Infection. N. Engl. J. Med..

[19] Lutgen V., Narasipura S.D., Barbian H.J., Richards M., Wallace J., Razmpour R., Buzhdygan T., Ramirez S.H., Prevedel L., Eugenin E.A. (2020). HIV infects astrocytes in vivo and egresses from the brain to the periphery. PLoS Pathog..

[20] Darcis G., Berkhout B., Pasternak A.O. (2019). The Quest for Cellular Markers of HIV Reservoirs: Any Color You Like. Front. Immunol..

[21] Descours B., Petitjean G., López-Zaragoza J.-L., Bruel T., Raffel R., Psomas C., Reynes C.P.J., Lacabaratz C., Levy Y., Schwartz T.B.O. (2017). CD32a is a marker of a CD4 T-cell HIV reservoir harbouring replication-competent proviruses. Nat. Cell Biol..

[22] Abdel-Mohsen M., Kuri-Cervantes L., Grau-Exposito J., Spivak A.M., Nell R.A., Tomescu C., Vadrevu S.K., Giron L.B., Serra-Peinado C., Genescà M. (2018). CD32 is expressed on cells with transcriptionally active HIV but does not enrich for HIV DNA in resting T cells. Sci. Transl. Med..

[23] Galvani A.P., Pandey A., Fitzpatrick M.C., Medlock J., Gray E.G. (2018). Defining control of HIV epidemics. Lancet HIV.

[24] Tyagi M., Bukrinsky M. (2012). Human Immunodeficiency Virus (HIV) Latency: The Major Hurdle in HIV Eradication. Mol. Med..

[25] Dash P.K., Kaminski R., Bella R., Su H., Mathews S., Ahooyi T.M., Chen C., Mancuso P., Sariyer R., Ferrante P. (2019). Sequential LASER ART and CRISPR Treatments Eliminate HIV-1 in a Subset of Infected Humanized Mice. Nat. Commun..

[26] Chun T.-W., Justement J.S., Murray D., Hallahan C.W., Maenza J., Collier A.C., Sheth P.M., Kaul R., Ostrowski M., Moir S. (2010). Rebound of plasma viremia following cessation of antiretroviral therapy despite profoundly low levels of HIV reservoir: Implications for eradication. AIDS.

[27] Deeks S.G., Lewin S.R., Ross A.L., Ananworanich J., Benkirane M., Cannon P., Chomont N., Douek D., Lifson J.D., International AIDS Society Towards a Cure Working Group International AIDS Society Towards a Cure Working Group (2016). International AIDS Society global scientific strategy: Towards an HIV cure. Nat. Med..

[28] Zhao J., Nguyen L.N.T., Dang X., Cao D., Khanal S., Schank M., Thakuri B.K.C., Ogbu S.C., Morrison Z.D., Wu X.Y. (2019). ATM Deficiency Accelerates DNA Damage, Telomere Erosion, and Premature T Cell Aging in HIV-Infected Individuals on Antiretroviral Therapy. Front. Immunol..

[29] Khanal S., Tang Q., Cao D., Zhao J., Nguyen L.N., Oyedeji O.S., Dang X., Schank M., Thakuri B.K.C., Ogbu C. (2020). Telomere and ATM Dynamics in CD4 T-Cell Depletion in Active and Virus-Suppressed HIV Infections. J. Virol..

[30] Bestilny L.J., Gill M.J., Mody C.H., Riabowol K.T. (2000). Accelerated replicative senescence of the peripheral immune system induced by HIV infection. AIDS.

[31] Gonzalez-Serna A., Ajaykumar A., Gadawski I., Muñoz-Fernández M.A., Hayashi K., Harrigan P.R., Côté H.C.F. (2017). Rapid Decrease in Peripheral Blood Mononucleated Cell Telomere Length After HIV Seroconversion, but Not HCV Seroconversion. JAIDS J. Acquir. Immune Defic. Syndr..

[32] Malaspina A., Moir S., Orsega S.M., Vasquez J., Miller N.J., Donoghue E.T., Kottilil S., Gezmu M., Follmann D., Vodeiko G.M. (2005). Compromised B Cell Responses to Influenza Vaccination in HIV-Infected Individuals. J. Infect. Dis..

[33] Rodriguez-Barradas M.C., Alexandraki I., Nazir T., Foltzer M., Musher D.M., Brown S., Thornby J. (2003). Response of Human Immunodeficiency Virus-Infected Patients Receiving Highly Active Antiretroviral Therapy to Vaccination with 23-Valent Pneumococcal Polysaccharide Vaccine. Clin. Infect. Dis..

[34] Patterson S.B., Landrum M.L., Okulicz J.F. (2014). Delayed-type hypersensitivity and hepatitis B vaccine responses, in vivo markers of cellular and humoral immune function, and the risk of AIDS or death. Vaccine.

[35] Balado M.D.M.D.P., Leal M., Lagares G.M., Mata R.C., López-Cortés L.F., Viciana P., Pacheco Y.M. (2010). Increased Regulatory T Cell Counts in HIV-Infected Nonresponders to Hepatitis B Virus Vaccine. J. Infect. Dis..

[36] Piekna-Przybylska D., Sharma G., Maggirwar S.B., Bambara R.A. (2017). Deficiency in DNA damage response, a new characteristic of cells infected with latent HIV. Cell Cycle.

[37] Van Der Watt G. (2011). Mitochondrial dysfunction and human immunodeficiency virus infection. J. Endocrinol. Metab. Diabetes S. Afr..

[38] Piekna-Przybylska D., Maggirwar S.B. (2018). CD4+ memory T cells infected with latent HIV-1 are susceptible to drugs targeting telomeres. Cell Cycle.

[39] Ji Y., Dang X., Nguyen L.N.T., Nguyen L.N., Zhao J., Cao D., Khanal S., Schank M., Wu X.Y., Morrison Z.D. (2019). Topological DNA damage, telomere attrition and T cell senescence during chronic viral infections. Immun. Ageing.

[40] Cao D., Zhao J., Nguyan L.N., Nguyen L.N.T., Khanal S., Dang X., Schank M., Thakuri B.K.C., Wu X.Y., Morrison Z.D. (2019). Disruption of Telomere Integrity and DNA Repair Machineries by KML001 Induces T Cell Senescence, Apoptosis, and Cellular Dysfunctions. Front. Immunol..

[41] Blanco J.-R., Jarrin I., Martinez A., Siles E., Larrayoz I.M., Cañuelo A., Gutierrez F., Gonzalez-Garcia J., Vidal F., Moreno S. (2015). Shorter Telomere Length Predicts Poorer Immunological Recovery in Virologically Suppressed HIV-1–Infected Patients Treated With Combined Antiretroviral Therapy. JAIDS J. Acquir. Immune Defic. Syndr..

[42] Leung J.M., Fishbane N., Jones M., Morin A., Xu S., Liu J.C., MacIsaac J., Milloy M.-J., Hayashi K., Montaner J. (2017). Longitudinal study of surrogate aging measures during human immunodeficiency virus seroconversion. Aging.

[43] Pathai S., Lawn S.D., Gilbert C.E., McGuinness D., McGlynn L., Weiss H.A., Port J., Christ T., Barclay K., Wood R. (2013). Accelerated biological ageing in HIV-infected individuals in South Africa. AIDS.

[44] Kirkwood T.B. (2005). Understanding the Odd Science of Aging. Cell.

[45] Tedone E., Huang E., O’Hara R., Batten K., Ludlow A.T., Lai T.-P., Arosio B., Mari D., Wright W.E., Shay J.W. (2018). Telomere length and telomerase activity in T cells are biomarkers of high-performing centenarians. Aging Cell.

[46] Boyle S.M., Fehr K., Deering C., Raza A., Harhay M.N., Malat G., Ranganna K., Lee D.H. (2020). Barriers to kidney transplant evaluation in HIV-positive patients with advanced kidney disease: A single-center study. Transpl. Infect. Dis..

[47] Ekat M.H., Diafouka M. (2015). Sp269antiretroviral Therapy-Related Nephrotoxicity in Hiv Infected Patients With Low Body Mass Index Outpatient Follow-Up In Brazzaville, Congo. Nephrol. Dial. Transplant..

[48] Ekat M.H., Ndour C.T., Ibara R.B.O., Diafouka M., Boumandoki P., Doukaga T.A., Aloumba G.A., Mahambou-Nsonde D., Nzounza P.R., Obengui P. (2020). Faible indice de masse corporelle et impact des antirétroviraux sur la néphrotoxicité, la maladie rénale chronique chez les patients infectés par le VIH à Brazzaville, Congo. Néphrologie Thérapeutique.

[49] McHugh G., Rehman A.M., Simms V., Gonzalez-Martinez C., Bandason T., Dauya E., Moyo B., Mujuru H., Rylance J., Sovershaeva E. (2020). Chronic lung disease in children and adolescents with HIV: A case—Control study. Trop. Med. Int. Heal..

[50] Toribio M., Neilan T.G., Zanni M.V. (2019). Heart Failure among People with HIV: Evolving Risks, Mechanisms, and Preventive Considerations. Curr. HIV/AIDS Rep..

[51] Barceló C., Guidi M., Thorball C.W., Hammer C., Chaouch A., Scherrer A.U., Hasse B., Cavassini M., Furrer H., Calmy A. (2020). Impact of Genetic and Nongenetic Factors on Body Mass Index and Waist-Hip Ratio Change in HIV-Infected Individuals Initiating Antiretroviral Therapy. Open Forum Infect. Dis..

[52] Shen J., Afaaf L., Shiau S., Strehlau R., Pierson S., Patel F., Wang L., Burke M., Violari A., Coovadia A. (2020). Mitochondrial Impairment in Well-Suppressed Children with Perinatal HIV-Infection on Antiretroviral Therapy. AIDS Res. Hum. Retroviruses.

[53] Erlandson K.M., Bradford Y., Samuels D.C., Brown T.T., Sun J., Wu K., Tassiopoulos K., Ritchie M.D., Haas D.W., Hulgan T. (2020). Mitochondrial DNA Haplogroups and Frailty in Adults Living with HIV. AIDS Res. Hum. Retrovir..

[54] Chwiki S., Campos M.M., McLaughlin M.E., Kleiner D.E., Kovacs J.A., Morse C.G., Abu-Asab M.S. (2017). Adverse effects of antiretroviral therapy on liver hepatocytes and endothelium in HIV patients: An ultrastructural perspective. Ultrastruct. Pathol..

[55] Yu F., Hao Y., Zhao H., Xiao J., Han N., Zhang Y., Dai G., Chong X., Zeng H., Zhang F. (2017). Distinct Mitochondrial Disturbance in CD4+T and CD8+T Cells From HIV-Infected Patients. JAIDS J. Acquir. Immune Defic. Syndr..

[56] Honnapurmath V.K., Patil V.W. (2017). Antiretroviral therapy-induced insulin resistance and oxidative deoxy nucleic acid damage in human immunodeficiency virus-1 patients. Indian J. Endocrinol. Metab..

[57] Wang G.P., Ciuffi A., Leipzig J., Berry C.C., Bushman F.D. (2007). HIV integration site selection: Analysis by massively parallel pyrosequencing reveals association with epigenetic modifications. Genome Res..

[58] Cohn L.B., Silva I.T., Oliveira T.Y., Rosales R.A., Parrish E.H., Learn G.H., Hahn B.H., Czartoski J.L., McElrath M.J., Lehmann C. (2015). HIV-1 Integration Landscape during Latent and Active Infection. Cell.

[59] Symons J., Chopra A., Malantinkova E., De Spiegelaere W., Leary S., Cooper D., Abana C.O., Rhodes A., Rezaei S.D., Vandekerckhove L. (2017). HIV integration sites in latently infected cell lines: Evidence of ongoing replication. Retrovirology.

[60] Brady T., Agosto L.M., Malani N., Berry C.C., O’Doherty U., Bushman F. (2009). HIV integration site distributions in resting and activated CD4 + T cells infected in culture. AIDS.

[61] Saleh S., Lu H.K., Evans V., Harisson D., Zhou J., Jaworowski A., Sallmann G., Cheong K.Y., Mota T.M., Tennakoon S. (2016). HIV integration and the establishment of latency in CCL19-treated resting CD4+ T cells require activation of NF-κB. Retrovirology.

[62] Einkauf K.B., Lee G.Q., Gao C., Sharaf R., Sun X., Hua S., Chen S.M., Jiang C., Lian X., Chowdhury F.Z. (2019). Intact HIV-1 proviruses accumulate at distinct chromosomal positions during prolonged antiretroviral therapy. J. Clin. Investig..

[63] Kim Y., Anderson J.L., Lewin S.R. (2018). Getting the “Kill” into “Shock and Kill”: Strategies to Eliminate Latent HIV. Cell Host Microbe.

[64] Cameron P.U., Saleh S., Sallmann G., Solomon A., Wightman F., Evans V.A., Boucher G., Haddad E.K., Sekaly R.-P., Harman A.N. (2010). Establishment of HIV-1 latency in resting CD4+ T cells depends on chemokine-induced changes in the actin cytoskeleton. Proc. Natl. Acad. Sci. USA.

[65] Martins L.J., Bonczkowski P., Spivak A.M., De Spiegelaere W., Novis C.L., DePaula-Silva A.B., Malatinkova E., Trypsteen W., Bosque A., Vanderkerckhove L. (2016). Modeling HIV-1 Latency in Primary T Cells Using a Replication-Competent Virus. AIDS Res. Hum. Retrovir..

[66] Bosque A., Planelles V. (2009). Induction of HIV-1 latency and reactivation in primary memory CD4+ T cells. Blood.

[67] Kula A., Delacourt N., Bouchat S., Darcis G., Avettand-Fenoel V., Verdikt R., Corazza F., Necsoi C., VanHulle C., Bendoumou M. (2019). Heterogeneous HIV-1 Reactivation Patterns of Disulfiram and Combined Disulfiram+Romidepsin Treatments. JAIDS J. Acquir. Immune Defic. Syndr..

[68] Cary D.C., Peterlin B.M. (2018). Procyanidin trimer C1 reactivates latent HIV as a triple combination therapy with kansui and JQ. PLoS ONE.

[69] Scheller C., Ullrich A., McPherson K., Hefele B., Knöferle J., Lamla S., Olbrich A.R., Stocker H., Arasteh K., Ter Meulen V. (2004). CpG Oligodeoxynucleotides Activate HIV Replication in Latently Infected Human T Cells. J. Biol. Chem..

[70] Huan C., Li Z., Ning S., Wang H., Yu X.-F., Zhang W. (2018). Long Noncoding RNA uc002yug.2 Activates HIV-1 Latency through Regulation of mRNA Levels of Various RUNX1 Isoforms and Increased Tat Expression. J. Virol..

[71] McBrien J.B., Mavigner M., Franchitti L., Smith S.A., White E., Tharp G.K., Walum H., Busman-Sahay K., Aguilera-Sandoval C.R., Thayer W.O. (2020). Robust and persistent reactivation of SIV and HIV by N-803 and depletion of CD8+ cells. Nat. Cell Biol..

[72] Nixon C.C., Mavigner M., Sampey G.C., Brooks A.D., Spagnuolo R.A., Irlbeck D.M., Mattingly C., Ho P.T., Schoof N., Cammon C.G. (2020). Systemic HIV and SIV latency reversal via non-canonical NF-κB signalling in vivo. Nat. Cell Biol..

[73] Perreau M., Savoye A.-L., De Crignis E., Corpataux J.-M., Cubas R., Haddad E.K., De Leval L., Graziosi C., Pantaleo G. (2013). Follicular helper T cells serve as the major CD4 T cell compartment for HIV-1 infection, replication, and production. J. Exp. Med..

[74] Banga R., Procopio F.A., Noto A., Pollakis G., Cavassini M., Ohmiti K., Corpataux J.-M., De Leval L., Pantaleo G., Perreau M. (2016). PD-1+ and follicular helper T cells are responsible for persistent HIV-1 transcription in treated aviremic individuals. Nat. Med..

[75] Noto A., Procopio F.A., Banga R., Suffiotti M., Corpataux J.M., Cavassini M., Riva A., Fenwick C., Gottardo R., Perreau M. (2018). CD32+ and PD-1+ lymph node CD4 T cells support persistent HIV-1 transcription in treated aviremic individuals. J. Virol..

[76] Fromentin R., DaFonseca S., Costiniuk C.T., El-Far M., Procopio F.A., Hecht F.M., Hoh R., Deeks S.G., Hazuda D.J., Lewin S.R. (2019). PD-1 blockade potentiates HIV latency reversal ex vivo in CD4+ T cells from ART-suppressed individuals. Nat. Commun..

[77] Kristoff J., Palma M.L., Garcia-Bates T.M., Shen C., Sluis-Cremer N., Gupta P., Rinaldo C.R., Mailliard R.B. (2019). Type 1-programmed dendritic cells drive antigen-specific latency reversal and immune elimination of persistent HIV. EBioMedicine.

[78] Rezaei S.D., Lu H.K., Chang J.J., Rhodes A., Lewin S.R., Cameron P.U. (2018). The Pathway To Establishing HIV Latency Is Critical to How Latency Is Maintained and Reversed. J. Virol..

[79] López-Huertas M.R., Morín M., Madrid-Elena N., Gutiérrez C., Jiménez-Tormo L., Santoyo J., Sanz-Rodríguez F., Pelayo M., Ángel M., Bermejo L.G. (2019). Selective miRNA Modulation Fails to Activate HIV Replication in In Vitro Latency Models. Mol. Ther. Nucleic Acids.

[80] Meås H.Z., Haug M., Beckwith M.S., Louet C., Ryan L., Hu Z., Landskron J., Nordbø S.A., Taskén K., Yin H. (2020). Sensing of HIV-1 by TLR8 activates human T cells and reverses latency. Nat. Commun..

[81] Grau-Expósito J., Luque-Ballesteros L., Navarro J., Curran A., Burgos J., Ribera E., Torrella A., Planas B., Badía R., Martin-Castillo M. (2019). Latency reversal agents affect differently the latent reservoir present in distinct CD4+ T subpopulations. PLoS Pathog..

[82] Serra-Peinado C., Grau-Expósito J., Luque-Ballesteros L., Astorga-Gamaza A., Navarro J., Gallego-Rodriguez J., Martin M., Curran A., Burgos J., Ribera E. (2019). Expression of CD20 after viral reactivation renders HIV-reservoir cells susceptible to Rituximab. Nat. Commun..

[83] Wilkinson B., Chen J.Y.-F., Han P., Rufner K.M., Goularte O.D., Kaye J. (2002). TOX: An HMG box protein implicated in the regulation of thymocyte selection. Nat. Immunol..

[84] Yun S., Lee S.H., Yoon S.-R., Kim M.S., Piao Z.-H., Myung P.-K., Kim T.-D., Jung H., Choi I. (2011). TOX regulates the differentiation of human natural killer cells from hematopoietic stem cells in vitro. Immunol. Lett..

[85] Aliahmad P., Kaye J. (2008). Development of all CD4 T lineages requires nuclear factor TOX. J. Exp. Med..

[86] Yu X., Li Z. (2015). TOX gene: A novel target for human cancer gene therapy. Am. J. Cancer Res..

[87] Dittmer S., Kovacs Z., Yuan S.H., Siszler G., Kögl M., Summer H., Geerts A., Golz S., Shioda T., Methner A. (2010). TOX3 is a neuronal survival factor that induces transcription depending on the presence of CITED1 or phosphorylated CREB in the transcriptionally active complex. J. Cell Sci..

[88] Du Puch C.B.M., Barbier E., Kraut A., Coute Y., Fuchs J., Buhot A., Livache T., Sève M., Favier A., Douki T. (2011). TOX4 and its binding partners recognize DNA adducts generated by platinum anticancer drugs. Arch. Biochem. Biophys..

[89] Vong Q.P., Leung W.-H., Houston J., Li Y., Rooney B., Holladay M., Oostendorp R.A.J., Leung W. (2014). TOX2 regulates human natural killer cell development by controlling T-BET expression. Blood.

[90] Seo H., Chen J., González-Avalos E., Samaniego-Castruita D., Das A., Wang Y.H., López-Moyado I.F., Georges R.O., Zhang W., Onodera A. (2019). TOX and TOX2 transcription factors cooperate with NR4A transcription factors to impose CD8+ T cell exhaustion. Proc. Natl. Acad. Sci. USA.

[91] Lobbardi R., Pinder J., Martinez-Pastor B., Theodorou M., Blackburn J.S., Abraham B.J., Namiki Y., Mansour M., Abdelfattah N.S., Molodtsov A. (2017). TOX Regulates Growth, DNA Repair, and Genomic Instability in T-cell Acute Lymphoblastic Leukemia. Cancer Discov..

[92] Morchikh M., Naughtin M., Di Nunzio F., Xavier J., Charneau P., Jacob Y., Lavigne M. (2013). TOX4 and NOVA1 Proteins Are Partners of the LEDGF PWWP Domain and Affect HIV-1 Replication. PLoS ONE.

[93] Gougeon M.-L., Melki M.-T., Saïdi H. (2011). HMGB1, an alarmin promoting HIV dissemination and latency in dendritic cells. Cell Death Differ..

[94] Naghavi M.H., Nowak P., Andersson J., Sönnerborg A., Yang H., Tracey K.J., Vahlne A. (2003). Intracellular high mobility group B1 protein (HMGB1) represses HIV-1 LTR-directed transcription in a promoter- and cell-specific manner. Virology.

[95] Nowak P., Abdurahman S., Lindkvist A., Troseid M., Sönnerborg A. (2012). Impact of HMGB1/TLR Ligand Complexes on HIV-1 Replication: Possible Role for Flagellin during HIV-1 Infection. Int. J. Microbiol..

[96] O’Flaherty E., Kaye J. (2003). TOX defines a conserved subfamily of HMG-box proteins. BMC Genom..

[97] Khan O., Giles J.R., McDonald S., Manne S., Ngiow S.F., Patel K.P., Werner M.T., Huang A.C., Alexander K.A., Wu J.E. (2019). TOX transcriptionally and epigenetically programs CD8+ T cell exhaustion. Nature.

[98] Agrawal V., Su M., Huang Y., Hsing M., Cherkasov A., Zhou Y. (2019). Computer-Aided Discovery of Small Molecule Inhibitors of Thymocyte Selection-Associated High Mobility Group Box Protein (TOX) as Potential Therapeutics for Cutaneous T-Cell Lymphomas. Molecules.

[99] Yang H., Wang H., Andersson U. (2020). Targeting Inflammation Driven by HMGB. Front. Immunol..

[100] Yang H., Wang H., Ju Z., Ragab A.A., Lundbäck P., Long W., Valdés-Ferrer S.I., He M., Pribis J.P., Li J. (2015). MD-2 is required for disulfide HMGB1&ndash, dependent TLR4 signaling. J. Exp. Med..

[101] Yang Y., Li S., Yang Q., Shi Y., Zheng M., Liu Y., Chen F., Song G., Xu H., Wan T. (2014). Resveratrol Reduces the Proinflammatory Effects and Lipopolysaccharide- Induced Expression of HMGB1 and TLR4 in RAW264.7 Cells. Cell. Physiol. Biochem..

[102] Vanpatten S., Al-Abed Y. (2018). High Mobility Group Box-1 (HMGb1): Current Wisdom and Advancement as a Potential Drug Target. J. Med. Chem..

[103] András I.E., Garcia-Contreras M., Yanick C., Perez P., Sewell B., Durand L., Toborek M. (2020). Extracellular vesicle-mediated amyloid transfer to neural progenitor cells: Implications for RAGE and HIV infection. Mol. Brain.

[104] Liu L., Yu J., Li L., Zhang B., Liu L., Wu C.-H., Jong A., Mao D.-A., Huang S.-H. (2017). Alpha7 nicotinic acetylcholine receptor is required for amyloid pathology in brain endothelial cells induced by Glycoprotein 120, methamphetamine and nicotine. Sci. Rep..

[105] Yuan S., Liu Z., Xu Z., Liu J., Zhang J. (2020). High mobility group box 1 (HMGB1): A pivotal regulator of hematopoietic malignancies. J. Hematol. Oncol..

[106] Stoszko M., Ne E., Abner E., Mahmoudi T. (2019). A broad drug arsenal to attack a strenuous latent HIV reservoir. Curr. Opin. Virol..

[107] Vargas B., Giacobbi N.S., Sanyal A., Venkatachari N.J., Han F., Gupta P., Sluis-Cremer N. (2018). Inhibitors of Signaling Pathways That Block Reversal of HIV-1 Latency. Antimicrob. Agents Chemother..

[108] Garcia-Vidal E., Badia R., Pujantell M., Castellví M., Felip E., Clotet B., Riveira-Muñoz E., Ballana E., Esté J.A. (2019). Dual effect of the broad spectrum kinase inhibitor midostaurin in acute and latent HIV-1 infection. Antivir. Res..

[109] Chougui G., Margottin-Goguet F. (2019). HUSH, a Link Between Intrinsic Immunity and HIV Latency. Front. Microbiol..

[110] Duverger A., Wolschendorf F., Anderson J.C., Wagner F., Bosque A., Shishido T., Jones J., Planelles V., Willey C., Cron R.Q. (2013). Kinase Control of Latent HIV-1 Infection: PIM-1 Kinase as a Major Contributor to HIV-1 Reactivation. J. Virol..

[111] Matsuda K., Kobayakawa T., Tsuchiya K., Hattori S.-I.H., Nomura W., Gatanaga H., Yoshimura K., Oka S., Endo Y., Tamamura H. (2019). Benzolactam-related compounds promote apoptosis of HIV-infected human cells via protein kinase C–induced HIV latency reversal. J. Biol. Chem..

[112] Van Montfort T., Van Der Sluis R., Darcis G., Beaty D., Groen K., Pasternak A.O., Pollakis G., Vink M., Westerhout E.M., Hamdi M. (2019). Dendritic cells potently purge latent HIV-1 beyond TCR-stimulation, activating the PI3K-Akt-mTOR pathway. EBioMedicine.

[113] Li C., Mousseau G., Valente S.T. (2019). Tat inhibition by didehydro-Cortistatin A promotes heterochromatin formation at the HIV-1 long terminal repeat. Epigenetics Chromatin.

[114] Besnard E., Hakre S., Kampmann M., Lim H.W., Hosmane N.N., Martin A., Bassik M.C., Verschueren E., Battivelli E., Chan J. (2016). The mTOR Complex Controls HIV Latency. Cell Host Microbe.

[115] Kulinski T., Olejniczak M., Huthoff H., Bielecki L., Pachulska-Wieczorek K., Das A.T., Berkhout B., Adamiak R.W. (2003). The Apical Loop of the HIV-1 TAR RNA Hairpin Is Stabilized by a Cross-loop Base Pair. J. Biol. Chem..

[116] Lu J., Kadakkuzha B.M., Zhao L., Fan M., Qi X., Xia T. (2011). Dynamic Ensemble View of the Conformational Landscape of HIV-1 TAR RNA and Allosteric Recognition. Biochemistry.

[117] Wei P., Garber E.M., Fang S.-M., Fischer W.H., Jones A.K. (1998). A Novel CDK9-Associated C-Type Cyclin Interacts Directly with HIV-1 Tat and Mediates Its High-Affinity, Loop-Specific Binding to TAR RNA. Cell.

[118] Garber M.E., Mayall T.P., Suess E.M., Meisenhelder J., Thompson N.E., Jones K.A. (2000). CDK9 Autophosphorylation Regulates High-Affinity Binding of the Human Immunodeficiency Virus Type 1 Tat–P-TEFb Complex to TAR RNA. Mol. Cell. Biol..

[119] Salerno D., Hasham M.G., Marshall R., Garriga J., Tsygankov A.Y., Graña X. (2007). Direct inhibition of CDK9 blocks HIV-1 replication without preventing T-cell activation in primary human peripheral blood lymphocytes. Gene.

[120] Mancebo H.S., Lee G., Flygare J., Tomassini J., Luu P., Zhu Y., Peng J., Blau C., Hazuda D., Price D. (1997). P-TEFb kinase is required for HIV Tat transcriptional activation in vivo and in vitro. Genes Dev..

[121] Yang X., Herrmann C.H., Rice A.P. (1996). The human immunodeficiency virus Tat proteins specifically associate with TAK in vivo and require the carboxyl-terminal domain of RNA polymerase II for function. J. Virol..

[122] Zhu Y., Pe’Ery T., Peng J., Ramanathan Y., Marshall N., Marshall T., Amendt B., Mathews M.B., Price D.H. (1997). Transcription elongation factor P-TEFb is required for HIV-1 Tat transactivation in vitro. Genes Dev..

[123] Budhiraja S., Famiglietti M., Bosque A., Planelles V., Rice A.P. (2013). Cyclin T1 and CDK9 T-Loop Phosphorylation Are Downregulated during Establishment of HIV-1 Latency in Primary Resting Memory CD4+ T Cells. J. Virol..

[124] Wang H., Song L., Zhou T., Zeng C., Jia Y., Zhao Y. (2020). A computational study of Tat–CDK9–Cyclin binding dynamics and its implication in transcription-dependent HIV latency. Phys. Chem. Chem. Phys..

[125] Morton E.L., Forst C.V., Zheng Y., DePaula-Silva A.B., Ramirez N.-G.P., Planelles V., D’Orso I. (2019). Transcriptional Circuit Fragility Influences HIV Proviral Fate. Cell Rep..

[126] Seif F., Khoshmirsafa M., Aazami H., Mohsenzadegan M., Sedighi G., Bahar M. (2017). The role of JAK-STAT signaling pathway and its regulators in the fate of T helper cells. Cell Commun. Signal..

[127] O’Shea J.J., Plenge R. (2012). JAK and STAT Signaling Molecules in Immunoregulation and Immune-Mediated Disease. Immunity.

[128] Rose N.R. (2017). Autoimmune Diseases. Int. Encycl. Public Health.

[129] Darnell J.E.D. (1997). STATs and Gene Regulation. Science.

[130] Quan Y., Xu H., Han Y., Mesplède T., Wainberg M.A. (2017). JAK-STAT Signaling Pathways and Inhibitors Affect Reversion of Envelope-Mutated HIV. J. Virol..

[131] Gavegnano C., Brehm J.H., Dupuy F.P., Talla A., Ribeiro S.P., Kulpa D.A., Cameron C., Santos S., Hurwitz S.J., Marconi V.C. (2017). Novel mechanisms to inhibit HIV reservoir seeding using Jak inhibitors. PLoS Pathog..

[132] Gavegnano C., Detorio M., Montero C., Bosque A., Planelles V., Schinazi R.F. (2014). Ruxolitinib and Tofacitinib Are Potent and Selective Inhibitors of HIV-1 Replication and Virus Reactivation In Vitro. Antimicrob. Agents Chemother..

[133] Venkatachari N.J., Zerbato J.M., Jain S., Mancini A.E., Chattopadhyay A., Sluis-Cremer N., Bar-Joseph Z., Ayyavoo V. (2015). Temporal transcriptional response to latency reversing agents identifies specific factors regulating HIV-1 viral transcriptional switch. Retrovirology.

[134] Bosque A., Nilson K.A., Macedo A.B., Spivak A.M., Archin N.M., Van Wagoner R.M., Martins L.J., Novis C.L., Szaniawski M.A., Ireland C.M. (2017). Benzotriazoles Reactivate Latent HIV-1 through Inactivation of STAT5 SUMOylation. Cell Rep..

[135] Selliah N., Zhang M., DeSimone D., Kim H., Brunner M., Ittenbach R.F., Rui H., Cron R.Q., Finkel T.H. (2006). The γc-cytokine regulated transcription factor, STAT5, increases HIV-1 production in primary CD4 T cells. Virology.

[136] Della Chiara G., Crotti A., Liboi E., Giacca M., Poli G., Lusic M. (2011). Negative Regulation of HIV-1 Transcription by a Heterodimeric NF-κB1/p50 and C-Terminally Truncated STAT5 Complex. J. Mol. Biol..

[137] Quan Y., Xu H., Kramer V.G., Han Y., Sloan R.D., Wainberg M.A. (2014). Identification of an env-defective HIV-1 mutant capable of spontaneous reversion to a wild-type phenotype in certain T-cell lines. Virol. J..

[138] Chaudhuri A., Yang B., Gendelman H.E., Persidsky Y., Kanmogne G.D. (2008). STAT1 signaling modulates HIV-1–induced inflammatory responses and leukocyte transmigration across the blood-brain barrier. Blood.

[139] Van Der Sluis R.M., Zerbato J.M., Rhodes J.W., Pascoe R.D., Solomon A., Kumar N.A., Dantanarayana A.I., Tennakoon S., Dufloo J., McMahon J. (2020). Diverse effects of interferon alpha on the establishment and reversal of HIV latency. PLoS Pathog..

[140] Gargan S., Ahmed S., Mahony R., Bannan C., Napoletano S., O’Farrelly C., Borrow P., Bergin C., Stevenson N.J. (2018). HIV-1 Promotes the Degradation of Components of the Type 1 IFN JAK/STAT Pathway and Blocks Anti-viral ISG Induction. EBioMedicine.

[141] Shytaj I.L., Lucic B., Forcato M., Penzo C., Billingsley J., Laketa V., Bosinger S., Stanic M., Gregoretti F., Antonelli L. (2020). Alterations of redox and iron metabolism accompany the development of HIV latency. EMBO J..

[142] Diaz R.S., Shytaj I.L., Giron L.B., Obermaier B., Della Libera E., Galinskas J., Dias D., Hunter J., Janini M., Gosuen G. (2019). Potential impact of the antirheumatic agent auranofin on proviral HIV-1 DNA in individuals under intensified antiretroviral therapy: Results from a randomised clinical trial. Int. J. Antimicrob. Agents.

[143] Valle-Casuso J.C., Angin M., Volant S., Passaes C., Monceaux V., Mikhailova A., Bourdic K., Avettand-Fenoel V., Boufassa F., Sitbon M. (2019). Cellular Metabolism Is a Major Determinant of HIV-1 Reservoir Seeding in CD4+ T Cells and Offers an Opportunity to Tackle Infection. Cell Metab..

[144] Castellano P., Prevedel L., Valdebenito S., Eugenin E.A. (2019). HIV infection and latency induce a unique metabolic signature in human macrophages. Sci. Rep..

[145] Thaker S.K., Ch’Ng J., Christofk H.R. (2019). Viral hijacking of cellular metabolism. BMC Biol..

[146] Eisenreich W., Rudel T., Heesemann J., Goebel W. (2019). How Viral and Intracellular Bacterial Pathogens Reprogram the Metabolism of Host Cells to Allow Their Intracellular Replication. Front. Cell. Infect. Microbiol..

[147] Timilsina U., Gaur R. (2016). Modulation of apoptosis and viral latency—an axis to be well understood for successful cure of human immunodeficiency virus. J. Gen. Virol..

[148] Bobardt M., Kuo J., Chatterji U., Chanda S., Little S.J., Wiedemann N., Vuagniaux G., Gallay P.A. (2019). The inhibitor apoptosis protein antagonist Debio 1143 Is an attractive HIV-1 latency reversal candidate. PLoS ONE.

[149] Bobardt M., Kuo J., Chatterji U., Wiedemann N., Vuagniaux G., Gallay P. (2020). The inhibitor of apoptosis proteins antagonist Debio 1143 promotes the PD-1 blockade-mediated HIV load reduction in blood and tissues of humanized mice. PLoS ONE.

[150] Kuo H.-H., Ahmad R., Lee G.Q., Gao C., Chen H.-R., Ouyang Z., Szucs M.J., Kim D., Tsibris A., Chun T.-W. (2018). Anti-apoptotic Protein BIRC5 Maintains Survival of HIV-1-Infected CD4+ T Cells. Immunity.

[151] Chugh P., Bradel-Tretheway B., Monteiro-Filho C.M., Planelles V., Maggirwar S.B., Dewhurst S., Kim B. (2008). Akt inhibitors as an HIV-1 infected macrophage-specific anti-viral therapy. Retrovirology.

[152] Wolf D., Witte V., Laffert B., Blume K., Stromer E., Trapp S., D’Aloja P., Schürmann A., Baur A.S. (2001). HIV-1 Nef associated PAK and PI3-Kinases stimulate Akt-independent Bad-phosphorylation to induce anti-apoptotic signals. Nat. Med..

[153] She Q.-B., Halilovic E., Ye Q., Zhen W., Shirasawa S., Sasazuki T., Solit D.B., Rosen N. (2010). 4E-BP1 Is a Key Effector of the Oncogenic Activation of the AKT and ERK Signaling Pathways that Integrates Their Function in Tumors. Cancer Cell.

[154] Rodrik-Outmezguine V.S., Chandarlapaty S., Pagano N.C., Poulikakos P.I., Scaltriti M., Moskatel E., Baselga J., Guichard S., Rosen N. (2011). mTOR Kinase Inhibition Causes Feedback-Dependent Biphasic Regulation of AKT Signaling. Cancer Discov..

[155] Campbell G.R., To R.K., Spector S.A. (2019). TREM-1 Protects HIV-1-Infected Macrophages from Apoptosis through Maintenance of Mitochondrial Function. mBio.

[156] Chandrasekar A.P., Cummins N.W., Badley A.D. (2019). The Role of the BCL-2 Family of Proteins in HIV-1 Pathogenesis and Persistence. Clin. Microbiol. Rev..

[157] Doitsh G., Galloway N.L.K., Geng X., Yang Z., Monroe K.M., Zepeda O., Hunt P.W., Hatano H., Sowinski S., Muñoz-Arias I. (2014). Cell death by pyroptosis drives CD4 T-cell depletion in HIV-1 infection. Nat. Cell Biol..

[158] Zhang G., Luk B.T., Wei X., Campbell G.R., Fang R.H., Zhang L., Spector S.A. (2019). Selective cell death of latently HIV-infected CD4+ T cells mediated by autosis inducing nanopeptides. Cell Death Dis..

[159] Cao J.Y., Dixon S.J. (2016). Mechanisms of ferroptosis. Cell. Mol. Life Sci..

[160] Wagner R.N., Reed J.C., Chanda S.K. (2015). HIV-1 protease cleaves the serine-threonine kinases RIPK1 and RIPK. Retrovirology.

[161] Fletcher-Etherington A., Nobre L., Nightingale K., Antrobus R., Nichols J., Davison A.J., Stanton R.J., Weekes M.P. (2020). Human cytomegalovirus protein pUL36: A dual cell death pathway inhibitor. Proc. Natl. Acad. Sci. USA.

[162] Mehrbod P., Ande S.R., Alizadeh J., Rahimizadeh S., Shariati A., Malek H., Hashemi M., Glover K.K.M., Sher A.A., Coombs K.M. (2019). The roles of apoptosis, autophagy and unfolded protein response in arbovirus, influenza virus, and HIV infections. Virulence.

[163] Swadling L., Pallett L.J., Diniz M.O., Baker J.M., Amin O.E., Stegmann K.A., Burton A.R., Schmidt N.M., Jeffery-Smith A., Zakeri N. (2020). Human Liver Memory CD8+ T Cells Use Autophagy for Tissue Residence. Cell Rep..

[164] He X., Yang W., Zeng Z., Wei Y., Gao J., Zhang B., Li L., Liu L., Wan Y., Zeng Q. (2020). NLRP3-dependent pyroptosis is required for HIV-1 gp120-induced neuropathology. Cell. Mol. Immunol..

[165] Carvalho A.R., Pinto C.M., Tavares J.N. (2019). Maintenance of the latent reservoir by pyroptosis and superinfection in a fractional order HIV transmission model. Int. J. Optim. Control. Theor. Appl..

[166] Narayanan A., Iordanskiy S., Das R., Van Duyne R., Santos S., Jaworski E., Guendel I., Sampey G., Dalby E., Iglesias-Ussel M. (2013). Exosomes Derived from HIV-1-infected Cells Contain Trans-activation Response Element RNA. J. Biol. Chem..

[167] Sampey G.C., Saifuddin M., Schwab A., Barclay R., Punya S., Chung M.-C., Hakami R.M., Zadeh M.A., Lepene B., Klase Z.A. (2016). Exosomes from HIV-1-infected Cells Stimulate Production of Pro-inflammatory Cytokines through Trans-activating Response (TAR) RNA. J. Biol. Chem..

[168] Henderson L.J., Johnson T.P., Smith B.R., Reoma L.B., Santamaria U.A., Bachani M., DeMarino C., Barclay R.A., Snow J., Sacktor N. (2019). Presence of Tat and transactivation response element in spinal fluid despite antiretroviral therapy. AIDS.

[169] Chen L., Feng Z., Yuan G., Emerson C.C., Stewart P.L., Ye F., Jin G. (2020). Human Immunodeficiency Virus-Associated Exosomes Promote Kaposi’s Sarcoma-Associated Herpesvirus Infection via the Epidermal Growth Factor Receptor. J. Virol..

[170] Barclay R.A., Schwab A., DeMarino C., Akpamagbo Y., Lepene B., Kassaye S., Iordanskiy S., Kashanchi F. (2017). Exosomes from uninfected cells activate transcription of latent HIV. J. Biol. Chem..

[171] Barclay R.A., Mensah G.A., Cowen M., DeMarino C., Kim Y., Pinto D.O., Erickson J., Kashanchi F. (2020). Extracellular Vesicle Activation of Latent HIV-1 Is Driven by EV-Associated c-Src and Cellular SRC-1 via the PI3K/AKT/mTOR Pathway. Viruses.

[172] Chen L., Feng Z., Yue H., Bazdar D., Mbonye U., Zender C., Harding C.V., Bruggeman L., Karn J., Sieg S.F. (2018). Exosomes derived from HIV-1-infected cells promote growth and progression of cancer via HIV TAR RNA. Nat. Commun..

[173] DeMarino C., Pleet M.L., Cowen M., Barclay R.A., Akpamagbo Y., Erickson J., Ndembi N., Charurat M., Jumare J., Bwala S. (2018). Antiretroviral Drugs Alter the Content of Extracellular Vesicles from HIV-1-Infected Cells. Sci. Rep..

[174] DeMarino C., Cowen M., Pleet M.L., Pinto D.O., Khatkar P., Erickson J., Docken S.S., Russell N., Reichmuth B., Phan T. (2020). Differences in Transcriptional Dynamics Between T-cells and Macrophages as Determined by a Three-State Mathematical Model. Sci. Rep..

[175] Sampey G.C., Iordanskiy S., Pleet M.L., DeMarino C., Romerio F., Mahieux R., Kashanchi F. (2020). Identification of Modulators of HIV-1 Proviral Transcription from a Library of FDA-Approved Pharmaceuticals. Viruses.

[176] Dobrowolski C., Valadkhan S., Graham A.C., Shukla M., Ciuffi A., Telenti A., Karn J., Ott M., Henderson A., Spina C. (2019). Entry of Polarized Effector Cells into Quiescence Forces HIV Latency. mBio.

[177] Theron A.J., Anderson R., Rossouw T.M., Steel H.C. (2017). The Role of Transforming Growth Factor Beta-1 in the Progression of HIV/AIDS and Development of Non-AIDS-Defining Fibrotic Disorders. Front. Immunol..

[178] Mbonye U., Wang B., Gokulrangan G., Shi W., Yang S., Karn J. (2018). Cyclin-dependent kinase 7 (CDK7)-mediated phosphorylation of the CDK9 activation loop promotes P-TEFb assembly with Tat and proviral HIV reactivation. J. Biol. Chem..

[179] Giffin M.J., Stroud J.C., Bates D.L., Von Koenig K.D., Hardin J.W., Chen L. (2003). Structure of NFAT1 bound as a dimer to the HIV-1 LTR κB element. Nat. Struct. Mol. Biol..

[180] Chan J.K., Bhattacharyya D., Lassen K.G., Ruelas D., Greene W.C. (2013). Calcium/Calcineurin Synergizes with Prostratin to Promote NF-κB Dependent Activation of Latent HIV. PLoS ONE.

[181] Gerritsen M.E., Williams A.J., Neish A.S., Moore S., Shi Y., Collins T. (1997). CREB-binding protein/p300 are transcriptional coactivators of p65. Proc. Natl. Acad. Sci. USA.

[182] Kinoshita S., Su L., Amano M., Timmerman A.L., Kaneshima H., Nolan G.P. (1997). The T Cell Activation Factor NF-ATc Positively Regulates HIV-1 Replication and Gene Expression in T Cells. Immunity.

[183] Yang X., Chen Y., Gabuzda D. (1999). ERK MAP Kinase Links Cytokine Signals to Activation of Latent HIV-1 Infection by Stimulating a Cooperative Interaction of AP-1 and NF-κB. J. Biol. Chem..

[184] Nabel G., Baltimore D. (1987). An inducible transcription factor activates expression of human immunodeficiency virus in T cells. Nat. Cell Biol..

[185] Mbonye U., Karn J. (2017). The Molecular Basis for Human Immunodeficiency Virus Latency. Annu. Rev. Virol..

[186] Cullen B.R., Lomedico P.T., Ju G. (1984). Transcriptional interference in avian retroviruses—Implications for the promoter insertion model of leukaemogenesis. Nat. Cell Biol..

[187] Lenasi T., Contreras X., Peterlin B.M. (2008). Transcriptional Interference Antagonizes Proviral Gene Expression to Promote HIV Latency. Cell Host Microbe.

[188] Colin L., Van Lint C. (2009). Molecular control of HIV-1 postintegration latency: Implications for the development of new therapeutic strategies. Retrovirology.

[189] Greger I.H. (1998). Transcriptional interference perturbs the binding of Sp1 to the HIV-1 promoter. Nucleic Acids Res..

[190] Renner D.B., Yamaguchi Y., Wada T., Handa H., Price D.H. (2001). A Highly Purified RNA Polymerase II Elongation Control System. J. Biol. Chem..

[191] Pagano J.M., Kwak H., Waters C.T., Sprouse R.O., White B.S., Ozer A., Szeto K., Shalloway D., Craighead H.G., Lis J.T. (2014). Defining NELF-E RNA Binding in HIV-1 and Promoter-Proximal Pause Regions. PLoS Genet..

[192] Kulkarni S., Lied A., Kulkarni V., Rucevic M., Martin M.P., Walker-Sperling V., Anderson S.K., Ewy R., Singh S., Nguyen H. (2019). CCR5AS lncRNA variation differentially regulates CCR5, influencing HIV disease outcome. Nat. Immunol..

[193] Rao S., Amorim R., Niu M., Breton Y., Tremblay M.J., Mouland A.J. (2019). Host mRNA decay proteins influence HIV-1 replication and viral gene expression in primary monocyte-derived macrophages. Retrovirology.

